# Dietary magnesium intake is related to larger brain volumes and lower white matter lesions with notable sex differences

**DOI:** 10.1007/s00394-023-03123-x

**Published:** 2023-03-10

**Authors:** Khawlah Alateeq, Erin I. Walsh, Nicolas Cherbuin

**Affiliations:** 1 National Centre for Epidemiology and Population Health, College of Health and Medicine, Australian National University, 54 Mills Road, Canberra, ACT 2601 Australia; 2 Radiological Science, College of Applied Medical Science, King Saud University, Riyadh, 11451 Saudi Arabia; 3 Population Health Exchange, National Centre for Epidemiology and Population Health, Australian National University, Canberra, Australia

**Keywords:** Magnesium, Neuroimaging, Blood pressure, Sex, UK biobank

## Abstract

**Purpose:**

To examine the association between dietary magnesium (Mg) intake and brain volumes and white matter lesions (WMLs) in middle to early old age.

**Methods:**

Participants (aged 40–73 years) from UK Biobank (*n* = 6001) were included and stratified by sex. Dietary Mg was measured using an online computerised 24 h recall questionnaire to estimate daily Mg intake. Latent class analysis and hierarchical linear regression models were performed to investigate the association between baseline dietary Mg, Mg trajectories, and brain volumes and WMLs. Associations between baseline Mg, and baseline blood pressure (BP) measures, and baseline Mg, Mg trajectories and BP changes (between baseline and wave 2) were also investigated to assess whether BP mediates the link between Mg intake and brain health. All analyses controlled for health and socio-demographic covariates. Possible interactions between menopausal status and Mg trajectories in predicting brain volumes and WMLs were also investigated.

**Results:**

On average, higher baseline dietary Mg intake was associated with larger brain volumes (gray matter [GM]: 0.001% [SE = 0.0003]; left hippocampus [LHC]: 0.0013% [SE = 0.0006]; and right hippocampus [RHC]: 0.0023% [SE = 0.0006]) in both men and women. Latent class analysis of Mg intake revealed three classes: “high-decreasing” (men = 3.2%, women = 1.9%), “low-increasing” (men = 1.09%, women = 1.62%), and “stable normal” (men = 95.71%, women = 96.51%). In women, only the “high-decreasing” trajectory was significantly associated with larger brain volumes (GM: 1.17%, [SE = 0.58]; and RHC: 2.79% [SE = 1.11]) compared to the “normal-stable”, the “low-increasing” trajectory was associated with smaller brain volumes (GM: − 1.67%, [SE = 0.30]; white matter [WM]: − 0.85% [SE = 0.42]; LHC: − 2.43% [SE = 0.59]; and RHC: − 1.50% [SE = 0.57]) and larger WMLs (1.6% [SE = 0.53]). Associations between Mg and BP measures were mostly non-significant. Furthermore, the observed neuroprotective effect of higher dietary Mg intake in the “high-decreasing” trajectory appears to be greater in post-menopausal than pre-menopausal women.

**Conclusions:**

Higher dietary Mg intake is related to better brain health in the general population, and particularly in women.

**Supplementary Information:**

The online version contains supplementary material available at 10.1007/s00394-023-03123-x.

## Introduction

The global prevalence of dementia is expected to rise dramatically from 57.4 million in 2019 to 152.8 million in 2050 [1]. This will impose a large burden on health and social services and incur considerable economic costs. Since there is no cure for dementia and the development of pharmacological treatment for dementia has been unsuccessful over the last 30 years, it has been suggested that greater attention should be directed towards prevention [2]. Several modifiable dementia risk factors have been identified but they currently only explain about 35% of the non-genetic risk [3]. There is, therefore, a pressing need to research and characterise factors contributing to the remaining unidentified risk. Emerging research suggests that dietary Magnesium (Mg) is associated with better cognitive function [4] and may reduce the risk of developing dementia [5]. However, it is unclear when dietary Mg intake starts contributing to brain health and in what way. This is a critical question since lifestyle and particularly diet are highly modifiable factors, and consequently could be promising targets for risk-reduction interventions in the population.

Mg has been implicated in the etiology and pathogenesis of various age-related brain disorders [6]. A high cerebral Mg level has been found to lower oxidative stress and systemic inflammation, enhance synaptic plasticity, and counteract other mechanisms that lead to neurodegeneration [6]. Moreover, a recent systematic review indicated that individuals with Alzheimer's disease (AD) have significant lower plasma Mg levels (− 0.89%) compared with healthy controls [7]. However, it should be noted that plasma Mg does not accurately reflect the body Mg content, because Mg is mainly stored in tissues with only 1% remaining in the blood. A large study investigating over 1000 middle-aged adults followed-up for 17 years showed that the highest quartile (≥ 196 mg/day) of dietary Mg intake is associated with a 37% reduced risk of developing dementia in elderly adults compared to the lowest quartile (≤ 174 mg/day) [5]. Consistent findings in individuals without dementia aged 60 or more suggest that higher dietary Mg intake (≥ 434 mg) is also linked to a lower risk of progressing from normal ageing to MCI (hazard ratios [HR] = 0.07, 95% confidence interval [CI] [0.01–0.56]) [8]. Furthermore, a recent study of 2507 cognitively healthy people aged ≥ 60 years found that dietary Mg intake (≥ 407 mg) was associated with higher global cognition (*β* = 0.15, 95% CI [0.2–0.28]), although this effect was only detected in women [4]. Together, these findings indicate that dietary Mg intake is implicated in biological processes related to brain ageing and may contribute to neurodegeneration in the pre-clinical stages leading to dementia. However, since most previous studies included participants above 60 years of age, it is unclear when the neuroprotective effects of dietary Mg become detectable.

Furthermore, dietary patterns are known to fluctuate, which may lead to changes in Mg intake over time. Such changes could be as or more influential than absolute Mg levels at any point in time as they may indicate changes in lifestyle or pathological processes. In this context, it is reasonable to assume that someone transitioning from low to high levels of Mg intake may not be comparable to someone with relatively stable Mg intake, even if their Mg intake is the same at one point in time. Conversely, recent changes, even if substantial, may not have had sufficient time to have an impact on processes implicated in brain ageing and neurodegeneration, and baseline levels may in some instances be more reflective of past long-term Mg intake. Therefore, it is important to contrast the effects of dietary Mg levels on brain ageing to those attributable to change in Mg intake as indexed by different trajectories.

The exact mechanisms underpinning Mg neuroprotective effects have not been clearly identified. There is considerable evidence that Mg levels are related to better cardiovascular health, and Mg supplementation has been found to decrease blood pressure (BP) [9], likely through its calcium antagonist action on smooth muscle tone, thus causing vasorelaxation [10]. Since high BP is a well-established risk factor for the dementia, decreasing its prevalence could significantly reduce age-related neurodegeneration and consequently lower the likelihood of developing dementia [11]. However, to our knowledge, no investigation of the link between the BP-lowering effect of Mg and neurodegeneration has been conducted.

It is important to note that other possible mechanisms may contribute to neuroprotective effects of Mg, although less evidence supporting their involvement is available. Neuroinflammation and the production of pro-inflammatory cytokines [12–14], and the regulation of the N-methyl-D-aspartate (NMDA) receptor excitability [15, 16] appear to be a strong alternative pathways which may be modulated by Mg. Together, these mechanisms could preserve cognitive function by increasing neurogenesis and decreasing neurodegeneration.

Given the current lack of understanding of when and to what extent dietary Mg exerts its neuroprotective effects on the brain and through which mechanisms, the aim of this study is to investigate associations between dietary Mg and brain volumes and white matter lesions (WMLs). Since diet is known to fluctuate over time [17], it stands to reason that dietary Mg may also change over time. This study will therefore also examine the association between Mg trajectories over time and brain volumes and WMLs. Furthermore, to determine whether BP mediates the link between Mg and neurodegeneration, and if so, which BP measures contribute most to this effect, this study will also examine the associations between Mg, and different BP measures (mean arterial pressure [MAP], SBP, DBP, pulse pressure [PP]), while also considering change in Mg and BP levels. Finally, because cardiovascular health, neurodegeneration, and brain shrinkage patterns differ between men and women [18–20], this study will investigate possible sex difference in these associations by stratifying analyses by sex.

## Methods

### Study design and participants

Participants included in the present study were selected from the UK biobank, which has been described elsewhere [21]. Briefly, the UK Biobank is a prospective cohort study of 502,655 participants aged 37–73 years at baseline who were assessed across 22 assessment centres around the UK between 2006 and 2023. Only those who had baseline DBP and SBP measurements (*n* = 456,990) and completed a structural magnetic resonance imaging (MRI) scan at the second evaluation (*n* = 36,260) were considered for inclusion. After excluding participants with neurological disorders (*n* = 3,275), and participants without dietary Mg intake data (*n* = 2,726), 6001 participants aged 40–73 years remained for analysis (Supplementary Fig. S1).

The UK Biobank Study received ethical approval from the North-West Multi-centre Research Ethics Committee (# 06/MRE08/65). All the participants provided informed consent to participate in the study. Further details of the UK Biobank's purpose, design, and survey methodologies have been published elsewhere [22]. This study follows the Strengthening the Reporting of Observational Studies in Epidemiology (STROBE) guidelines [23].

### Blood pressure

Blood pressure was assessed as the average of two measurements of brachial SBP/DBP on an Omron M4 monitor (OMRON Healthcare Europe, NA, Hoofddorp) in seated position, using a well-fitted cuff following a 5-min rest. The participants’ MAP and PP were calculated using the formulas: MAP mmHg = DBP + (1/3 × [SBP –DBP]) and PP mmHg: (SBP–DBP). Hypertension was defined by SBP/DBP ≥ 140/90 mmHg, or use of antihypertensive medication [24].

### Dietary Mg intake

Dietary Mg intake was measured using the Oxford WebQ, a computerized 24 h recall questionnaire self-completed online [25, 26]. This questionnaire was designed to be completed multiple times to minimise measurement error that could occur with a single 24 h recall assessment. The questionnaire consists of 200 foods with a range of portion sizes. Nutrient intake of Mg was computed using data from McCance and Widdowson's “The Composition of Food and its Supplements”, further details of its methodology have been described elsewhere [26]. Examples of foods which were assessed and contain higher Mg levels include leafy green vegetables such as spinach, legumes, nuts, seeds and whole grains. Participants were invited to take the Oxford WebQ at five time points over a 16-month period, varying weekdays, weekends, and seasonality. It has been validated against an interviewer-administered 24 h recall, with only minor discrepancies in nutrient consumption reported using both approaches [25, 26].

### Image acquisition

Participants underwent a MRI scan during the second (2014 +) visits at one of three imaging centres using the same scanner (3 T Siemens Skyra, running VD13A SP4 using a 32-channel head coil). Detailed imaging protocols are provided online (http://biobank.ctsu.ox.ac.uk/crystal/refer.cgi?id=1977) [21].

Briefly, all participants were imaged with a T1-weighted 3D magnetization-prepared rapid acquisition gradient echo sequence over a five-minute duration in the sagittal orientation (resolution = 1 × 1 × 1 mm; matrix size: 208 × 256 × 256).

### Segmentation and image analysis

FreeSurfer was used to segment and analyse neuroimaging data (version 6.0.5) [27]. The FreeSurfer pipeline has been extensively described elsewhere [28]. In summary, it involves motion correction, transformation to Talairach image space, inhomogeneity correction, non-brain tissue removal using hybrid watershed, volumetric segmentation [29, 30], and cortical surface reconstruction and parcellation [21, 31]. Regions of interest (ROIs) considered a priori in the analysis were total gray matter (GM), white matter (WM), left (LHC) and right hippocampus (RHC), and white matter lesions (WMLs). These ROIs were selected because they have been linked to high BP and brain ageing in previous studies [20, 32].

### Covariates

Covariates included age, sex, BMI, serum high-density lipoprotein (HDL), total cholesterol (TC), education level, diabetes mellitus diagnosed by a doctor, self-reported smoking status (i.e., never, previous, or current); physical activity (i.e., the total amount of time spent in vigorous, moderate, and mild activity weighted by the amount of energy expended in each of these categories of activity, METs/week), and alcohol intake (drinks/week).

### Statistical analyses

Statistical analyses were computed using the R statistical package (Version 1.2.5019) under Rstudio (Version 1.2.5019) [33]. Group differences were assessed using t-tests for continuous measures and chi-square tests for categorical data. The distribution of each variable was examined, and skewed data were transformed using log transformations. Mg intake was centered on 350 mg (recommended daily intake ~ 310–420) to facilitate interpretation [34].

### Trajectories

Latent class analysis (LCA) was used to identify mutually exclusive subgroup (classes) of Mg intake trajectory separately for men and women [35]. This approach enables the identification of distinct trajectories taken by subgroups of individuals who follow similar progression of dietary Mg intake over time [1]. The trajectories can then be interpreted and labelled (e.g., stable, increasing, decreasing, etc.) and used to determine whether certain patterns of Mg intake relate to different outcomes (e.g., impaired cognition, lower brain volumes). The “lcmm” package (version 1.7.5) [36] was used to determine the appropriate number of classes (2, 3, 4, or 5).

Latent classes were modelled using a linear link function with the number of waves as the time predictor. The best fitting latent class sets were selected based on the model Akaike information criterion (AIC) [37], the Bayesian information criterion (BIC) [38], stable allocation of participants to the classes (> 50% of members of each class with posterior probability > 0.7), and at least 20 participants in each class [19]. Participants’ characteristic in each class was examined using Wilcoxon signed-rank, or Fisher's exact tests (Supplementary Tables S3 & S4).

### Generalised linear models

Hierarchical linear regression models were applied to assess the association between 1) baseline Mg, Mg trajectories and brain volumes (GM, WM, LHC, RHC, WMLs) at wave 2; 2) baseline Mg and baseline BP (MAP, SBP, DBP and PP); and 3) baseline Mg, Mg trajectories and BP changes (Δ) between baseline and wave 2.

Three main models were fit. Model 1 was controlled for age and education. Model 2 additionally tested the two-way interactions between baseline Mg and Mg trajectories, while controlling for antihypertensive medication. Model 3 additionally controlled for cardiovascular risk factors including diabetes mellitus, HDL, TC, alcohol intake, physical activity, smoking status, and BMI. All analyses were stratified by sex as prior research has demonstrated sex variation in brain ageing [19] and BP [25], and because pilot analyses indicated significant interactions between baseline Mg, Mg trajectories and sex. Moreover, previous studies have shown a significant neuroprotective effect of estrogen which may explain some of the sex differences observed in the main analyses [39]. Therefore, we additionally tested the two-way interactions between self-reported menopausal status and Mg levels/trajectories in women while controlling for age, education and cardiovascular risk factors including diabetes mellitus, HDL, TC, alcohol intake, physical activity, smoking status, and BMI. (Model 3).

The unstandardized beta coefficient, standard error, and p-values for outcomes measures are reported. The significance threshold was set at *p* < 0.05 and corrected for multiple comparisons (Bonferroni).

## Results

### Participant characteristics

Participant characteristics are presented in Table 1. Men were slightly older (~ 1 year) and had slightly higher Mg intake (~ 30–40 mg), BMI (+ 1.2 ${\mathrm{kg}/\mathrm{m}}^{2}$), SBP (+ 6.5 mmHg), and DBP (+ 3.84 mmHg), and had a higher prevalence of BP medication (+ 1.8%) and diabetes mellitus (+ 1.6%), than women. The differences in the demographic characteristics between men and women were mostly non-significant except for the BP medication, and diabetes mellitus (> 0.5) which were slightly higher in men than women. Women characteristics split by menopausal status are presented in Table S12. Postmenopausal women had slightly higher Mg intake (~ 5–10 mg), BMI (+ 0.54 ${\mathrm{kg}/\mathrm{m}}^{2}$), SBP (+ 9.5 mmHg), and DBP (+ 1.5 mmHg), and had a higher prevalence of BP medication (+ 3.8%) and diabetes mellitus (+ 1.24%), than pre-menopausal women. However, the differences in the demographic characteristics between pre- and post-menopausal women were mostly non-significant.

**Table 1:** Participants’ demographic characteristics

Measures	Whole sample	Men	Women	T/chi-sq test (*P* value)
Age, year (SD)	55.33 (7.47)	56.01 (7.55)	54.70 (7.34)	6.80 (0.000)
Magnesium (time 1), mg (SD)	361.66 (125.52)	383.06 (132.19)	342.34 (115.85)	12.63 (0.000)
Magnesium (time 2), mg (SD)	354.46 (97.55)	372.62 (102.39)	338.08 (89.89)	13.82 (0.000)
Magnesium (time 3), mg (SD)	355.57 (96.81)	376.99 (100.98)	336.23 (88.56)	16.55 (0.000)
Magnesium (time 4), mg (SD)	350.48 (100.13)	367.80 (103.01)	334.85 (94.78)	12.85 (0.000)
Magnesium (time 5), mg (SD)	354.56 (99.68)	372.50 (106.12)	338.38 (90.51)	13.33 (0.000)
GM volume, mm^3^ (SD)	668,596.78 (59,511.14)	702,602.25 (53,509.78)	637,901.28 (46,564.76)	49.72 (0.000)
WM volume, mm^3^ (SD)	479,863.85 (57,394.42)	510,092.93 (54,011.60)	452,577.18 (45,446.94)	44.38 (0.000)
LHC volume, mm^3^ (SD)	3695.06 (396.08)	3828.33 (401.37)	3574.77 (350.16)	25.95 (0.000)
RHC volume, mm^3^ (SD)	3810.40 (403.18)	3950.41 (409.80)	3684.01 (352.23)	26.87 (0.000)
WMLs, mm^3^ (SD)	7.37 (0.66)	7.50 (0.66)	7.26 (0.64)	14.76 (0.000)
ICV, mm^3^ (SD)	1,553,957.98 (151,384.80)	1,643,664.98 (133,929.80)	1,472,982.76 (116,595.32)	52.40 (0.000)
SBP, mmHg (SD)	134.71 (17.84)	138.17 (16.57)	131.58 (18.37)	14.61 (0.000)
DBP, mmHg (SD)	81.12 (9.96)	83.14 (9.88)	79.30 (9.68)	15.17 (0.000)
MAP, mmHg (SD)	98.98 (11.77)	101.48 (11.33)	96.73 (11.70)	15.98 (0.000)
PP, mmHg (SD)	53.59 (12.33)	55.04 (11.28)	52.28 (13.06)	8.76 (0.000)
BMI, kg/m^2^ (SD)	26.51 (4.14)	27.09 (3.76)	25.99 (4.40)	10.51 (0.000)
Cholesterol, mmol/L (SD)	5.75 (1.07)	5.62 (1.07)	5.86 (1.06)	-8.66 (0.000)
HDL, mmol/L (SD)	1.49 (0.36)	1.32 (0.29)	1.64 (0.36)	-39.16 (0.000)
Hypertension, *n* (%)	2430 (40.41%)	1355 (47.69%)	1063 (33.77%)	119.74 (0.000)
BP medication, *n* (%)	444 (7.40%)	237 (8.32%)	207 (6.56%)	6.52 (0.011)
Diabetes mellitus,* n* (%)	167 (2.78%)	99 (3.48%)	68 (2.16%)	9.17 (0.002)
Ever smoked, *n* (%)	3711 (61.84%)	1641 (57.64%)	2070 (65.63%)	40.16 (0.000)
Higher education, *n* (%)	2957 (49.28%)	1477 (51.88%)	1480 (46.92%)	14.50 (0.000)

Significance: *p* < 0.05. Abbreviations: *GM* gray matter, *WM* white matter, *LHC* lift hippocampus, *RHC* right hippocampus, *WMLs* white matter lesions, *ICV* intracranial volume, *SBP* systolic blood pressure, *DBP* diastolic blood pressure, *MAP* mean arterial pressure, *PP* pulse pressure, *BMI* body mass index, *HDL* high-density lipoprotein, *BP* blood pressure

Pearson bivariate correlations between covariates are presented in Fig. S2. Most relationships between variables of interest were small to moderate, except among BP measurements (> 0.91).

### Latent class analysis

**Fig. 1:**
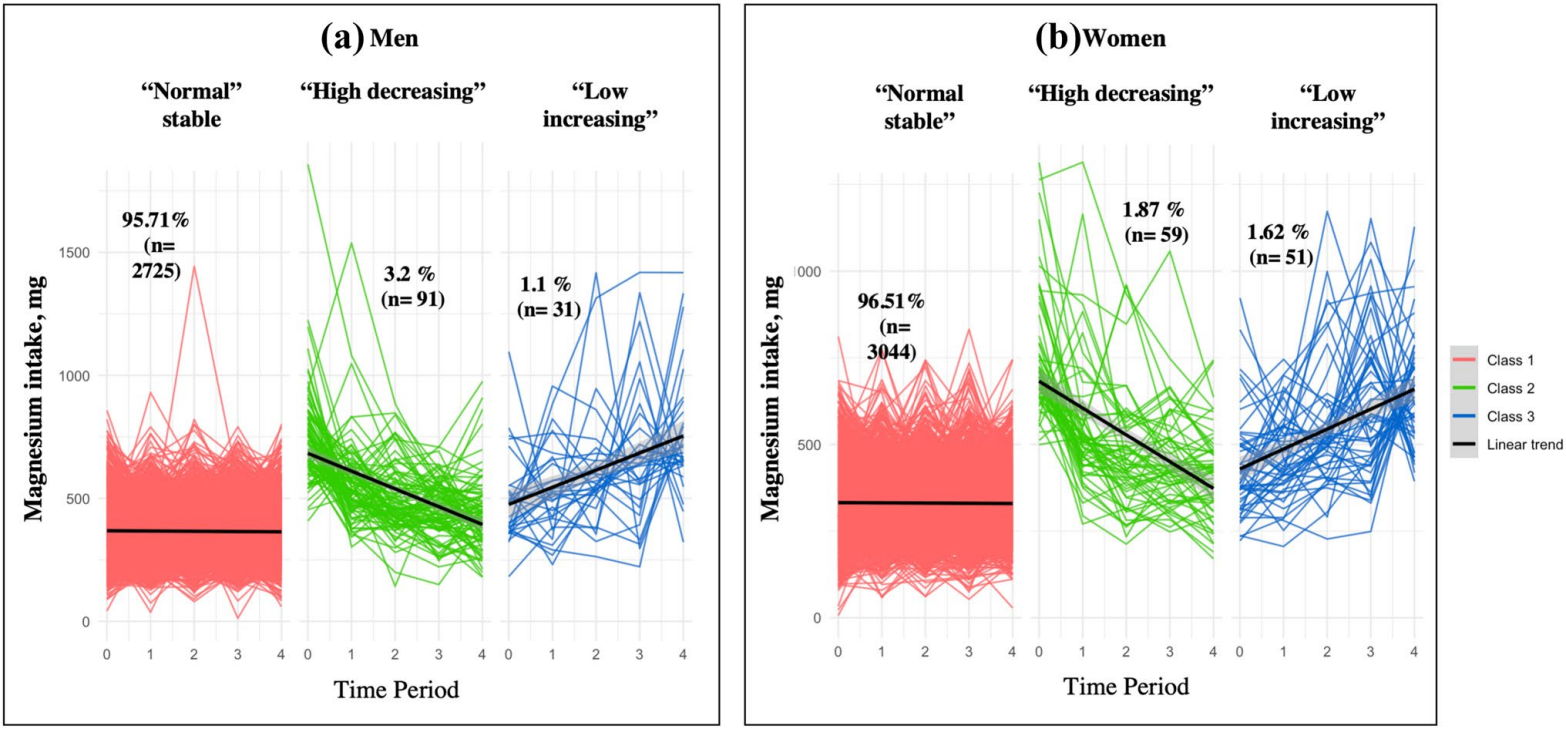
Dietary Mg intake trajectories in men and women corresponding to different subgroups of individuals identified by latent class analysis. Note: “Time” is the number of waves across 16 months. Line intercept and slope are drawn from a linear model for each trajectory, with time as only predictor of Mg intake. Black lines represent the mean trend in each class. Coloured lines (red, green, or blue) correspond to the number of participants per class, and the slopes represent the change in Mg per unit of time. “n” is the number of participants in each class. The exploratory modelling and the Bootstrap Likelihood Ratio Test indicated that a three-class solution was optimal: class1 (“normal-stable”), class 2 (“high-decreasing”), and class 3 (“low-increasing”)

**Fig. 2:**
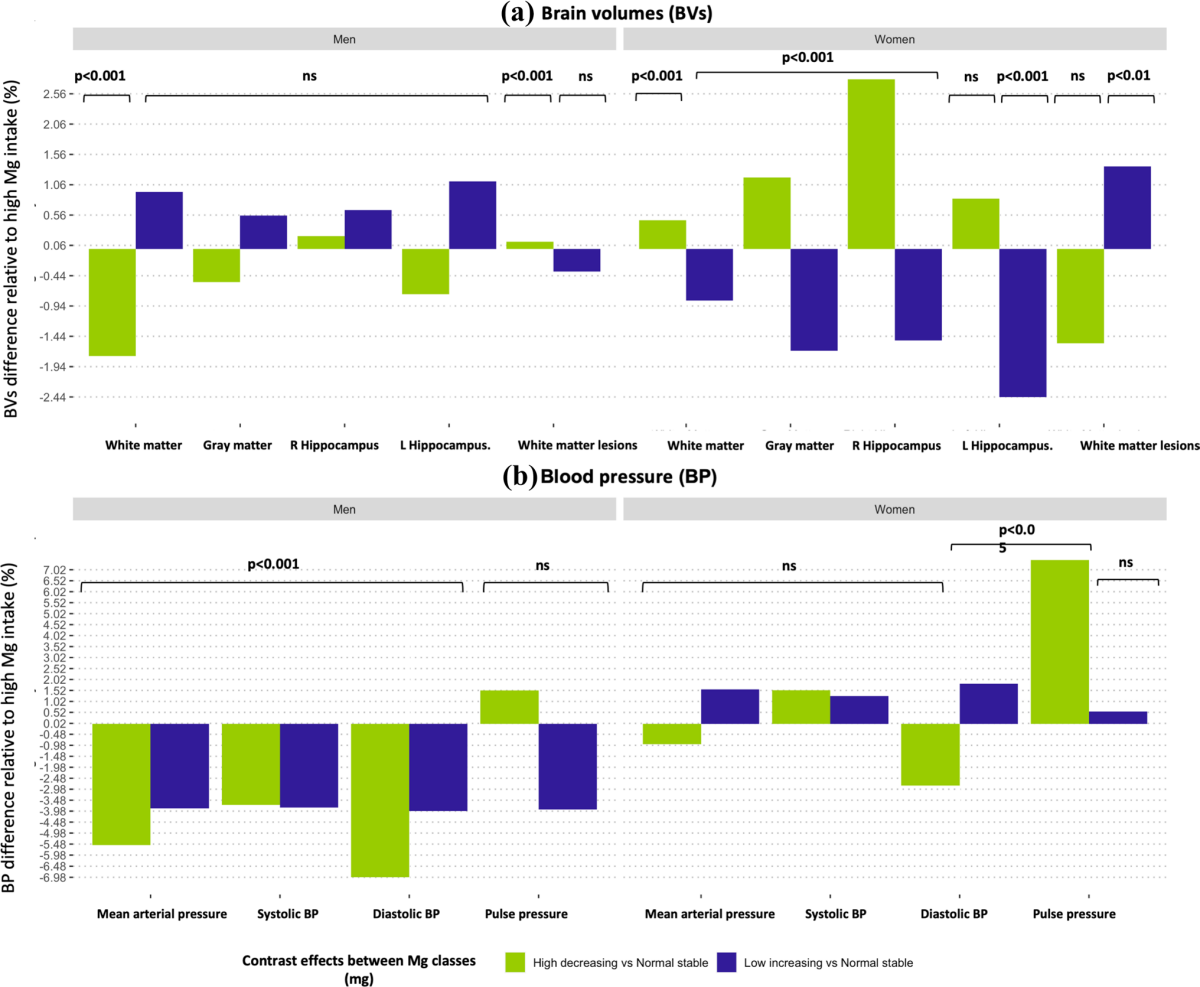
Bar graph of the associations (beta values) between dietary magnesium (Mg) trajectories and **a** the brain volumes including gray matter, white matter, left hippocampus, right hippocampus, and white matter lesions; and **b** blood pressure (BP) including mean arterial pressure (MAP), systolic blood pressure (SBP), diastolic blood pressure (DBP), pulse pressure (PP) stratified by sex

Models with up to five classes were estimated and assessed based on model fit, stability, and class sizes. The best fit was obtained for a three-class model both in men and women (Table S2). The corresponding trajectories were interpreted as “normal-stable” (men = 95.71%, women = 96.51%), “high-decreasing” (men = 3.2%, women = 1.9%), and “low-increasing” (men = 1.09%, women = 1.62%) (Fig. 1). Mg levels in each class and across waves are presented in Tables S3 & S4. On average, they tended to be higher in men than women but followed similar trajectories in the two sexes (Fig. 2).

### Baseline Mg, Mg trajectories, and brain volumes

Associations between baseline Mg, Mg trajectories, and brain volumes (wave 2) are presented in Table 2. Higher baseline Mg levels were strongly associated with larger brain volumes (except for WMLs for which the association was reversed), with some variation across ROIs and sexes. Every 1 mg higher in baseline Mg above 350 mg/day was associated with a 0.0011% larger GM, − 0.0011% smaller WM, 0.0008% larger LHC, 0.0023% RHC, and − 0.001% lower WMLs in men, and with a 0.001% larger GM, 0.001% larger WM, 0.0018% larger LHC, 0.0023% RHC, and 0.0012 larger WMLs in women.

**Table 2:** Associations between baseline Mg, Mg trajectories and brain volumes and white matter lesions

	Gray matter volume (mm^3^)		White matter volume (mm^3^)		Left hippocampal volume (mm^3^)		Right hippocampal volume (mm^3^)		White matter lesions volume (mm^3^)	
	Men	Women	Men	Women	Men	Women	Men	Women	Men	Women
	Beta (SE)	Beta (SE)	Beta (SE)	Beta (SE)	Beta (SE)	Beta (SE)	Beta (SE)	Beta (SE)	Beta (SE)	Beta (SE)
“High-decreasing”	– 3,845.243 (3,256.911)	7,521.830^**^ (3,722.368)	– 9,284.898^***^ (3,319.663)	2,185.547 (3,770.945)	– 28.579 (34.672)	29.757 (40.965)	8.319 (35.498)	103.124^**^ (40.895)	0.009 (0.057)	– 0.113 (0.076)
“low-increasing”	3,874.717 (3,127.947)	– 10,706.060^****^ (1,922.555)	4,950.949 (3,188.214)	– 3,945.020^**^ (1,947.644)	42.825 (33.299)	– 87.213^****^ (21.158)	25.388 (34.092)	-55.489^***^ (21.122)	– 0.028 (0.055)	0.099^**^ (0.039)
Baseline Mg	8.308^****^ (2.420)	6.875^***^ (2.196)	– 5.286^**^ (2.466)	2.998 (2.225)	0.032 (0.026)	0.065^***^ (0.024)	0.092^****^ (0.026)	0.086^****^ (0.024)	– 0.0001^***^ (0.00004)	0.0001^**^ (0.00004)
Constant	340,933.300^****^ (4,794.676)	243,385.100^****^ (3,723.059)	108,298.000^****^ (4,887.057)	39,506.150^****^ (3,771.645)	2,828.207^****^ (51.042)	2,417.771^****^ (40.973)	2,807.002^****^ (52.258)	2,303.468^****^ (40.903)	2.463^****^ (0.085)	2.914^****^ (0.076)

Significance. *p < 0.05; **p < 0.01; ***p < 0.001. Abbreviation; *SE* standard error, *Mg* magnesium. Note. Hierarchical analysis results of the association between baseline Mg intake, trajectories (“High-decreasing” vs “normal-stable” and “low-increasing” vs” normal-stable”) and the brain volumes including gray matter, white matter left hippocampus right hippocampus and white matter lesions. Model 1 was adjusted for the main covariates including age, and education. Model 2 was additionally tested the two-way interactions between baseline Mg x Mg trajectories while controlling for baseline Mg and antihypertensive medication. Model 3 was additionally adjusted for the other covariates: high-density lipoprotein (HDL), cholesterol, diabetes mellitus, smoking status, higher education, physical activity, and alcohol intake. Data represents unstandardized Beta correlation ± SE. Beta is per 1 mg unit increment in Mg intake variables and expressed in SD units of the dependent variable

Moreover, the “high-decreasing” trajectory was associated with larger brain volumes when compared to the “normal-stable” trajectory. The association was significant for GM and HC, and more so in women than men. Every 1 mg higher Mg above 350 mg/day was associated with a − 0.54% smaller GM, and 0.21% larger RHC in men, and with a 1.17% larger GM, and 2.79% larger RHC in women. However, these associations did not reach significance in men.

Furthermore, the “low-increasing” trajectory was associated with smaller brain volumes when compared to the “normal-stable” trajectory (except for the WMLs for which the association was reversed). The association was significant for GM, WM, HC, and WMLs, and more so in women than men. Every 1 mg higher in Mg above 350 mg/day was associated with a 0.55% larger GM, 0.93% larger WM, 1.11% larger LHC, 0.64% larger RHC, and − 0.37% smaller WMLs in men, and with a − 1.67% smaller GM, − 0.85% smaller WM, − 2.43% smaller LHC, − 1.50% smaller RHC, and 1.6% larger WMLs in women. However, these associations did not reach significance in men.

Two-way interactions between Mg and Mg trajectories as predictors of brain volumes and WMLs are presented in Tables S5, S7, and Fig. S3. No consistent pattern was detected but significant interactions were observed between baseline Mg, Mg trajectory, and GM, HC and WMLs, and more so in women than men. The “high-decreasing” trajectory was significantly associated with smaller GM and RHC and larger WMLs in women. The “low-increasing” trajectory was significantly associated with larger GM and smaller WMLs in women.

### Baseline Mg and baseline BP

Associations between baseline (wave 1) Mg and baseline BP are presented in Table S8. Baseline Mg levels were not significantly associated with baseline BP measures, except for PP for which every 1 mg higher Mg above 350 mg/day was associated with a 0.001% higher PP in men, and 0.004% higher PP in women.

### Baseline Mg, Mg trajectories and BP changes

Associations between baseline Mg, Mg trajectories and BP changes (between baseline and wave 2) are presented in Table 3. Higher baseline Mg levels were not significantly associated with BP changes across BP types and sexes.

**Table 3:** Associations between baseline Mg, Mg trajectories and blood pressure changes (between baseline and wave 2)

	Δ MAP (mmHg)		Δ SBP (mmHg)		Δ DBP (mmHg)		Δ PP (mmHg)	
	Men	Women	Men	Women	Men	Women	Men	Women
	Beta (SE)	Beta (SE)	Beta (SE)	Beta (SE)	Beta (SE)	Beta (SE)	Beta (SE)	Beta (SE)
“High-decreasing”	– 5.599^****^ (1.120)	– 0.898 (1.735)	– 5.097^***^ (1.686)	2.013 (2.703)	– 5.802^****^ (1.004)	– 2.234 (1.506)	0.833 (1.276)	3.902^*^ (2.057)
“Low-increasing”	– 3.913^****^ (1.154)	1.521^*^ (0.878)	– 5.274^***^ (1.737)	1.661 (1.367)	– 3.308^***^ (1.035)	1.449^*^ (0.762)	– 2.149 (1.315)	0.293 (1.040)
Baseline Mg	0.001 (0.001)	– 0.001 (0.001)	0.002 (0.001)	– 0.002 (0.001)	0.0004 (0.001)	– 0.001 (0.001)	0.001 (0.001)	– 0.001 (0.001)
Constant	46.831^****^ (1.411)	36.473^****^ (1.159)	46.455^****^ (2.078)	40.089^****^ (1.728)	45.868^****^ (1.263)	32.436^****^ (1.032)	1.205 (1.493)	3.828^***^ (1.258)

Significance. *p < 0.05; **p < 0.01; ***p < 0.001. Abbreviations: *SE* standard error, *MAP* mean arterial pressure, *SBP* systolic blood pressure, *DBP* diastolic blood pressure, *PP* pulse pressure, *Mg* magnesium; Δ: changes. Note: Hierarchical analysis results of the association between baseline Mg intake, trajectories (“high-decreasing” vs “normal-stable” and “low-increasing” vs “normal-stable”) and ΔBP (ΔMAP, ΔSBP, ΔDBP, ΔPP). Model 1 was adjusted for baseline BP and the main covariates including age, and education. Model 2 was additionally tested the two-way interactions between baseline Mg x Mg trajectories while controlling for baseline Mg and antihypertensive medication. Model 3 was additionally adjusted for main covariates: high-density lipoprotein (HDL), cholesterol, diabetes mellitus, smoking status, higher education, physical activity, and alcohol intake. Data represents unstandardized Beta correlation ± SE. Beta is per 1 mg unit increment in Mg intake variables and expressed in SD units of the dependent variable

However, the “high-decreasing” trajectory was associated with a decreasing MAP (between baseline and wave 2) when compared to the “normal-stable” trajectory. Moreover, the effect was greater (~ 3.07%) for DBP than SBP, and more so in men than women. Every 1 mg lower Mg below 350 mg/day was associated with a − 6.97% decrease DBP, and − 3.68% decrease SBP in men, and − 2.81% decrease DBP, and a 2.05% increase SBP in women. However, these associations did not reach significance in women.

Also, a significant association between “high-decreasing” trajectory and PP was detected but only in women. Every 1 mg lower Mg below 350 mg/day was associated with a 1.51% increase PP in men, and a 7.4% increase PP in women.

Moreover, the “low-increasing” trajectory was associated with a decreasing MAP (between baseline and wave 2) when compared to the “normal-stable” trajectory. The effect of Mg was greater (~ 0.21%) for DBP than SBP, and more so in men than women. Every additional 1 mg Mg above 350 mg/day was associated with a − 3.97% decrease DBP, and − 3.81% decrease SBP in men, and with a 1.81% increase DBP, and a non-significant 1.26% increase SBP in women.

Two-way interactions between baseline Mg, and Mg trajectories as predictors of BP changes (between baseline and wave 2) are presented in Tables S9 & S11, and Fig. S4. Significant interactions between baseline Mg, Mg trajectories and MAP, SBP, and DBP were detected, and more so in men than women. The “high-decreasing” trajectory was more highly associated with increased MAP, SBP and DBP.

### Effects of menopause

Detailed analyses of menopausal status and its interactions with Mg trajectories are reported in Table 4. Menopausal status was found to significantly modulate the association between Mg trajectories and brain volumes and WMLs with some notable differences between trajectories and ROIs.

**Table 4:** Associations between baseline Mg, Mg trajectories and brain volumes and white matter lesions in pre- and post-menopausal women

	Gray matter volume (mm^3^)	White matter volume (mm^3^)	Left hippocampal volume (mm^3^)	Right hippocampal volume (mm^3^)	White matter lesions volume (mm^3^)
	Beta (SE)	Beta (SE)	Beta (SE)	Beta (SE)	Beta (SE)
High decreasing	14,783.680^****^ (4,444.086)	– 7,184.026^**^ (2,876.470)	– 31.966 (31.275)	83.701^*^ (48.777)	0.030 (0.090)
Low increasing	– 11,085.040^****^ (2,897.717)	– 8,480.628^***^ (2,807.397)	– 70.208^**^ (30.524)	– 22.470 (31.804)	– 0.067 (0.059)
Baseline Mg	7.193^***^ (2.195)	3.214 (2.113)	0.066^***^ (0.023)	0.091^****^ (0.024)	0.0001^*^ (0.00004)
Menopause	3,108.057^****^ (658.623)	5,086.465^****^ (665.678)	56.997^****^ (7.238)	54.778^****^ (7.229)	– 0.127^****^ (0.013)
Menopause x High decreasing	– 9,738.396^***^ (3,285.059)	16,174.570^****^ (3,315.210)	37.365 (36.045)	27.388 (36.056)	– 0.195^***^ (0.067)
Menopause x low increasing	781.198 (3,449.958)	4,874.314 (3,480.971)	– 21.366 (37.848)	– 49.109 (37.866)	0.256^****^ (0.070)
Constant	249,069.800^****^ (3,933.080)	50,326.560^****^ (3,976.134)	2,530.299^****^ (43.232)	2,410.303^****^ (43.168)	2.665^****^ (0.080)

Significance. *p < 0.05; **p < 0.01; ***p < 0.001. Abbreviation; *SE* standard error, *Mg* magnesium. Note. Hierarchical analysis results of the association between baseline Mg intake, trajectories (“High-decreasing” vs “normal-stable” and “low-increasing” vs” normal-stable”) and the brain volumes including gray matter, white matter left hippocampus right hippocampus and white matter lesions. Model 1 was adjusted for the main covariates including age, and education. Model 2 was additionally tested the two-way interactions between baseline Mg x Mg trajectories while controlling for baseline Mg and antihypertensive medication. Model 3 was additionally tested the two-way interactions between menopause status x Mg trajectories while adjusted for the other covariates: high-density lipoprotein (HDL), cholesterol, diabetes mellitus, smoking status, higher education, physical activity, and alcohol intake**.** Data represents unstandardized Beta correlation ± SE. Beta is per 1 mg unit increment in Mg intake variables and expressed in SD units of the dependent variable

Higher baseline Mg levels were more strongly associated with larger brain volumes after menopause (except for WMLs for which the association was reversed). Every additional 1 mg Mg above 350 mg/day was associated with a 0.4% larger GM, 1.09% larger WM, 1.5% larger LHC, 1.4% RHC, and − 1.7% lower WMLs in post-menopausal compared to pre-menopausal women.

Similarly, post-menopausal women in the “high-decreasing” trajectory had significantly larger WM volumes and lower WMLs than pre-menopausal women. Every additional 1 mg Mg above 350 mg/day was associated with a 3.4% larger WM and –2.6% lower WMLs in post-menopausal compared to pre-menopausal women.

In contrast, post-menopausal women in the “low-increasing” trajectory had significantly larger WMLs than pre-menopausal women. Every additional 1 mg Mg above 350 mg/day was associated with a 3.5% larger WMLs in post-menopausal compared to pre-menopausal women. However, there were no significant interaction between Mg trajectories and menopausal status for HC.

## Discussion

This study offers some important findings indicating that (1) higher dietary Mg is associated with larger brain volumes and lower WMLs; (2) dietary Mg effects differ by sex and are more evident in women; and (3) higher dietary Mg is generally not associated with lower BP in this population.

A novel result is that dietary Mg was associated with larger brain volumes and lower WMLs, indicating better brain health. This association was found in cross-sectional analyses, but importantly was also detected when contrasting Mg trajectories reflecting changes in Mg levels in three distinct groups of people who could be interpreted as “normal-stable”, “high-decreasing”, and “low-increasing”. The current application of trajectory analysis provides more nuanced insights by considering both current Mg intake and change in Mg intake over time. In cross-sectional analyses, higher dietary Mg intake was related to larger brain volumes and lower WMLs in a variety of brain regions, including GM, WM, and HC. Alterations in these regions are likely to have substantial implications for brain ageing. The GM and HC are composed of a large number of neurons, allowing them to process information which is then shared between brain regions through axon signalling in the WM. These processes allow individuals to plan, communicate and regulate their movements, memories, and emotions. Over time, structural changes and tissue loss reflected by lower brain volumes are likely to increasingly impact cognitive function and impair the individual’s capacity to function. Moreover, the progression of WMLs is important since they affect cognitive performance across all domains [40]. A somewhat unexpected result when considering the differences in associations with brain volume between the three trajectories identified is that the “high-decreasing” trajectory was found to be associated with larger brain volumes than the “normal-stable” trajectory. It might have been predicted that a decrease in Mg levels, and hence decreasing neuroprotective effects, would be associated with lower brain volumes. Although we cannot be sure of the reason for this result, a possible interpretation is that the high baseline dietary Mg intake level may be more reflective of habitual dietary patterns preceding the study window in participants from this group. This explanation would be consistent with the lower brain volumes detected in those with “low-increasing” Mg levels. Thus, we hypothesise that the long-term exposure to dietary Mg, over years or decades, which is known to be relatively stable [41–43], underlies the observed difference in brain ageing. This is also consistent with studies showing that underlying neuropathological mechanisms develop over long periods and often during or before mid-life [44].

It is noteworthy that the seemingly neuroprotective effect of Mg was substantial and varied across brain regions. It was particularly strong for GM and HC. Indeed, our models indicate that compared to somebody with a normal Mg intake (~ 350 mg/day), somebody in the top quartile of Mg intake (≥ 550 mg/day) would be predicted to have a ~ 0.20% larger GM and ~ 0.46% larger RHC. In a population with an average age of 55 years this effect corresponds to ~ 1 year of typical ageing [45–50]. In other words, if this effect is generalisable to other populations, a 41% increase in Mg intake may lead to significantly better brain health, which would also be expected to contribute to greater preservation of cognitive ability, and lower risk or delayed onset of dementia in later life. Indeed, a high dietary Mg intake (≥ 196 mg/day) has been found to be associated with a 37% reduced future risk of dementia in individuals in their 60s. This is an important result, especially in light of recent research in AD animal models. Li et al. [51] observed that rats supplemented with Mg (in drinking water) had less synaptic loss, fewer amyloid plaques in HC, and improved learning and memory compared to controls. This is consistent with the beneficial effect suggested by our findings which may also have important implications for the development AD pathology. Moreover, since our findings were demonstrated in mid-life individuals, they suggest that dietary Mg intake may contribute to neuroprotection earlier in the ageing process and that preventative effects may begin in the 40s or even before. Importantly, these effects were observed in a general population with normal Mg levels, indicating that they are not due to clinical Mg deficiency. Also, while we are unaware of any previous study linking dietary Mg and brain structure in humans, our results are consistent with evidence linking green vegetable intake—a known source of Mg—and larger GM and WM volumes [52].

The effects of some of the mechanisms likely to underpin this study's findings have been demonstrated by research in mice showing that Mg deficiency is linked to microglia activation and the production of pro-inflammatory cytokines including interleukin 1 beta (IL-1β), IL-6 and Tumor Necrosis Factor (TNF) [12]. In contrast, Mg supplementation has an anti-inflammatory effect by decreasing neuroinflammatory mediators [53, 54], which in turn increases neurogenesis by up-regulating the proliferation of neuronal stem cells (NSC) in the HC. Furthermore, these effects persisted in aged mice [55], may together lead to reduced neurodegeneration. However, in human, these effects develop over decades and their impact on brain health may not be detected until later life. This is consistent with the Ozawa et al. [5] and Lo et al.’s [16] findings which demonstrated an association between middle-age dietary Mg intake and the development of dementia 17 to 20 years later.

Another important finding is that while the cross-sectional analyses showed strong associations between dietary Mg and brain volumes and WMLs in men and women, the possible neuroprotective effects of Mg trajectories only reached significance in women. Previous research has indicated that low Mg serum levels are associated with impaired cognitive function in women but not in men [56]. In the current study, the sex difference is not due to differences in Mg intake, as men had slightly higher baseline Mg intakes than women and followed similar trajectories. However, it is possible that changes in estrogen levels related to menopause contribute to the protective effect of Mg in women and explain some of the sex differences. Thus, we conducted additional analysis to test the interaction effects between Mg intake and menopause status in predicting brain volumes. We predicted that the strength of association between higher dietary Mg intake and larger brain volumes would be greater in pre-menopausal women, because Mg makes positive contributes to the regulation and maintenance of healthy estrogen levels in the body [57] and is also important for preventing hypertension [9], improving insulin sensitivity, and reducing cardio-metabolic risk factors﻿﻿ [58, 59]. However, this was not supported by our results which showed a stronger association between Mg intake and brain volumes in post-menopausal women across several brain regions suggesting a greater neuroprotective effect of Mg intake. This effect may be attributable to the anti-inflammatory effect of Mg. Indeed, Chacko et al.'s [60] study demonstrated significantly lower systemic inflammatory markers such as c-reactive protein (CRP), TNF, and IL-6 in post-menopausal women with higher dietary Mg intake. Since neuroinflammation tends to increase with increasing age and the observed concurrent increase in cardio-metabolic risk factors, it is possible that post-menopausal women “benefit” more from Mg’s anti-inflammatory effect than pre-menopausal women, who as a group tend to have lower inflammation levels. Also consistent with these findings, greater brain volume differences were detected between the “high-decreasing” trajectory and the “normal-stable” trajectory in post-menopausal women suggesting that higher Mg intake may have a greater protective effect in post-menopausal women. However, little evidence of an effect of menopause was found when comparing the “low increasing” and “stable normal” trajectories. This may indicate that relatively little differences in Mg intake exist between these trajectories in pre- and post-menopausal women. Together, these findings suggest a greater neuroprotective effect of dietary Mg intake in post-menopausal women, however, further research is required to confirm and clarify this relationship. In addition, a number of health conditions, whose incidence varies between men and women, may also influence the Mg associations in men and women [61]. Thus, it is likely that sex effects are the result of combinations of causes that are beyond the scope of this study, but which should be investigated in future research.

A particularly unexpected finding is that, while our working hypothesis was that any association between dietary Mg and brain volumes would be mediated by an effect on BP, not only did we not find any evidence of such a mediation effect, but there was also little evidence of any association between Mg intake and BP, except for PP. This suggests that alternative mechanisms may be involved. A plausible mechanism is inflammation. Mg plays an essential role in the regulation of oxidative stress and neuroinflammation [6, 62]. In an AD mice model, Mg deficiency increased the production of a neuroinflammatory tachykinin which stimulate the secretion of pro-inflammatory mediators and nitric oxide [53]. Since there is accumulating evidence indicating that oxidative stress and neuroinflammation are major contributors to neurodegeneration and AD [13, 63], their possible role in mediating the effect of Mg requires more attention in future research. Another mechanism that also warrants consideration is Mg’s role in blocking the cytotoxic effects of NMDA [15].

This study has not only a number of limitations but also significant strengths. Mg intake was measured indirectly (i.e. food frequency questionnaires), which is known to be reliable [64] but to be also associated with greater measurement noise and, therefore, may have decreased our capacity to detect associations. However, diet was measured multiple times during the follow-up period, which minimizes any recall bias effect [64], and accounts for changes in habitual dietary intake [25]. While we carefully adjusted for the influence of several important cardiovascular, lifestyle, and socio-demographic risk factors in our analyses, it is likely that we did not fully account for their contribution or the influence of other uncontrolled risk factors on brain morphology in men and women. Hence, future research should attempt to include and compare a wider range of health conditions to reach a more accurate understanding of how Mg intake differentially influences brain health in men and women. A distinct strength is that this study investigated a very large sample with sufficient statistical power to examine hypothesised associations while controlling for a large number of covariates. To our knowledge, it was also the first to use neuroimaging and structural brain measures in humans to examine the effect of dietary Mg intake. Furthermore, individuals with neurological disorders were excluded to reduce the possibility that the effects detected were driven by clinical neuropathology.

## Conclusion

In summary, this study provides new evidence that higher dietary Mg intake is related to better brain health in the general population. Since dietary advice and supplementation are easily scalable further research on the benefits of dietary Mg needs to be conducted to provide the necessary evidence base to support possible population health interventions aimed at mitigating age-related neurodegeneration.

## Supplementary Information

Below is the link to the electronic supplementary material.

Supplementary file1 (DOCX 20264 KB)

## Data Availability

UK Biobank is an open access resource accessible to confirmed researchers upon request (ukbiobank.ac.uk/).
