# Supplementary material for: Dietary magnesium intake is related to larger brain volumes and lower white matter lesions with notable sex differences
Source: Eur J Nutr. 2023 Mar 10;62(5):2039–51. doi: 10.1007/s00394-023-03123-x (PMC10349698; doi:10.1007/s00394-023-03123-x)
Supplement: Supplementary file 1 — Supplementary file1 (DOCX 20264 KB) [file 394_2023_3123_MOESM1_ESM.docx]

**Title:** The relationship between Blood pressure, magnesium, and Cerebral Health

**Authors:** Ms. Khawlah Alateeq, MMagReson^1,2*,^ Dr. Erin Walsh, PhD^1,^ , Prof. Nicolas Cherbuin, PhD^1^

**Affiliations :** Centre for Research on Ageing, Health and Wellbeing, Australian National University, Canberra, Australia.^1^ , King Saud University, Riyadh, Saudi Arabia.^2^

**Figure S1.** Selection of study population resulting in a total sample of 6001 individuals of the total UK Biobank cohort.

Participant data provided as part of application number

(n = 502,537)

**Identification**

Participants with systolic and diastolic Blood pressure measures at baseline (2006-2010)
(n = 456,990)

**Screening**

Participants excluded due to neurological disorder including dementia, Alzheimer disease, epilepsy, Parkinson’s disease, multiple sclerosis, or stroke

(n =3,275)

Participants without confounding diagnoses
(n =453715)

Participants with available FreeSurfac brain imaging data
(n =36,260)

**Eligibility**

Total cases excluded
(n = 466,277)

Study population first project
(n = 36,260)

Participants with dietary magnesium intake at baseline data

(n = 6001)

**Included**

Total cases excluded
(n = 496,536)

Study population
(n = 6001)

| **Table S1**. Selected vs non-selected | | | | |
| --- | --- | --- | --- | --- |
|  | | | | |
| Measures | Whole Sample | Not selected | Selected | T/chi-sq Test (P value) |
|  | | | | |
| Age, year (SD) | 56.53 (8.10) | 56.43 (8.09) | 56.54 (8.10) | -2.81 (0.005) |
| SBP, mmHg (SD) | 139.74 (19.70) | 139.97 (20.35) | 139.73 (19.68) | 1.20 (0.230) |
| DBP, mmHg (SD) | 82.21 (10.70) | 82.66 (11.19) | 82.19 (10.69) | 4.36 (0.000) |
| BMI, kg/m2 (SD) | 27.43 (4.80) | 27.77 (5.21) | 27.40 (4.76) | 14.40 (0.000) |
| Cholesterol, mmol/L | 5.69 (1.14) | 5.70 (1.16) | 5.69 (1.14) | 1.14 (0.255) |
| HDL mmol/L | 1.45 (0.38) | 1.43 (0.38) | 1.45 (0.38) | -7.35 (0.000) |
| Significance: p<0.05 | | | | |

There were (0.05%) participants missing data for body weight index (BMI), (5.7%) participants missing data for cholesterol, (14.4%) participants missing data for HDL, (%0.02) participants missing data for diabetes, (0.04%) participants missing data for alcohol conception, (0.2%) participants missing data for smoking status, 335 (0.9%), participants missing data for antihypertensive medication, and (14.2%) participants missing data for physical activity. All missing values were imputed.


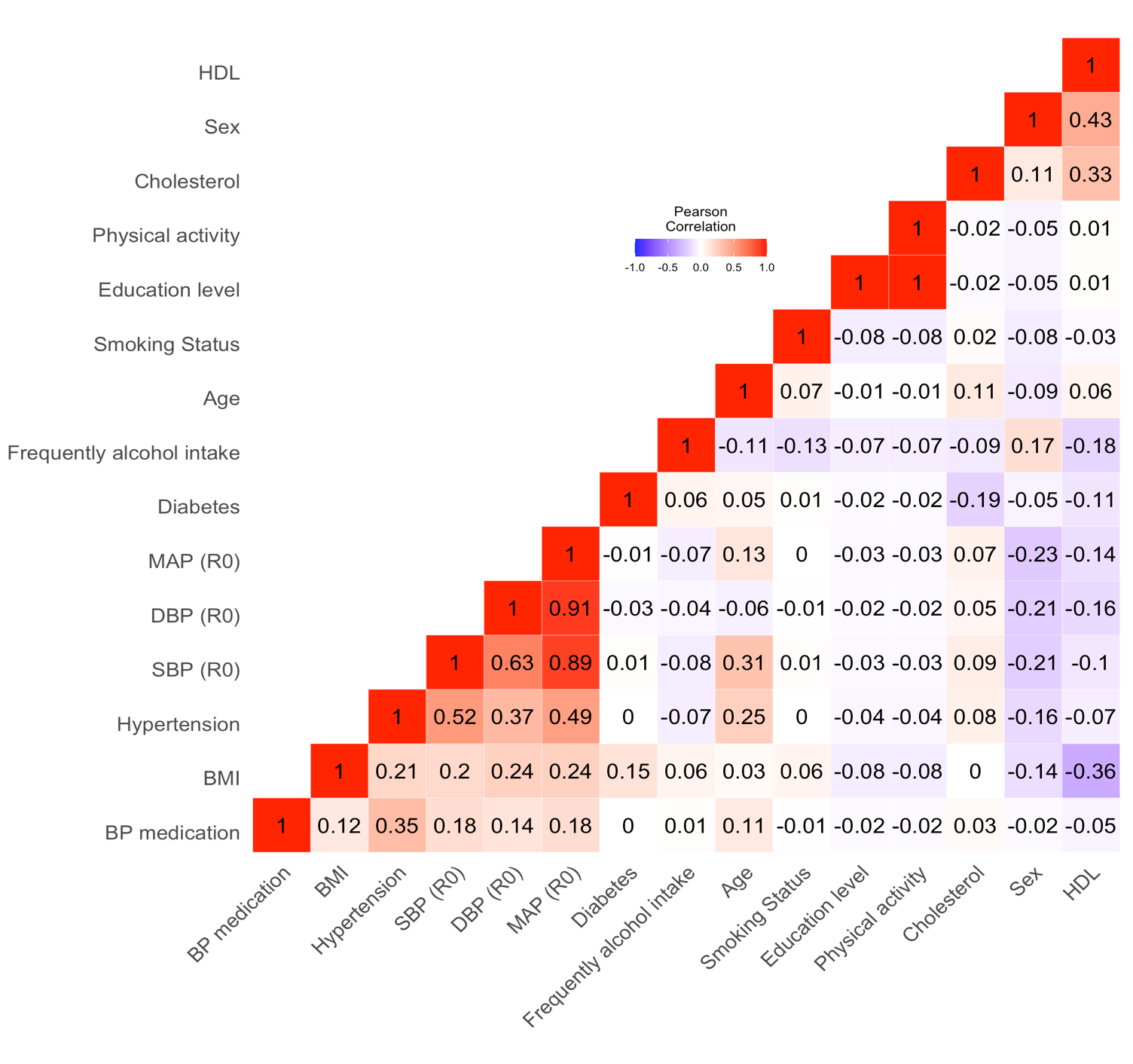


**Figure S2**. Bivariate correlation matrix between BP measurements and main covariates.

**
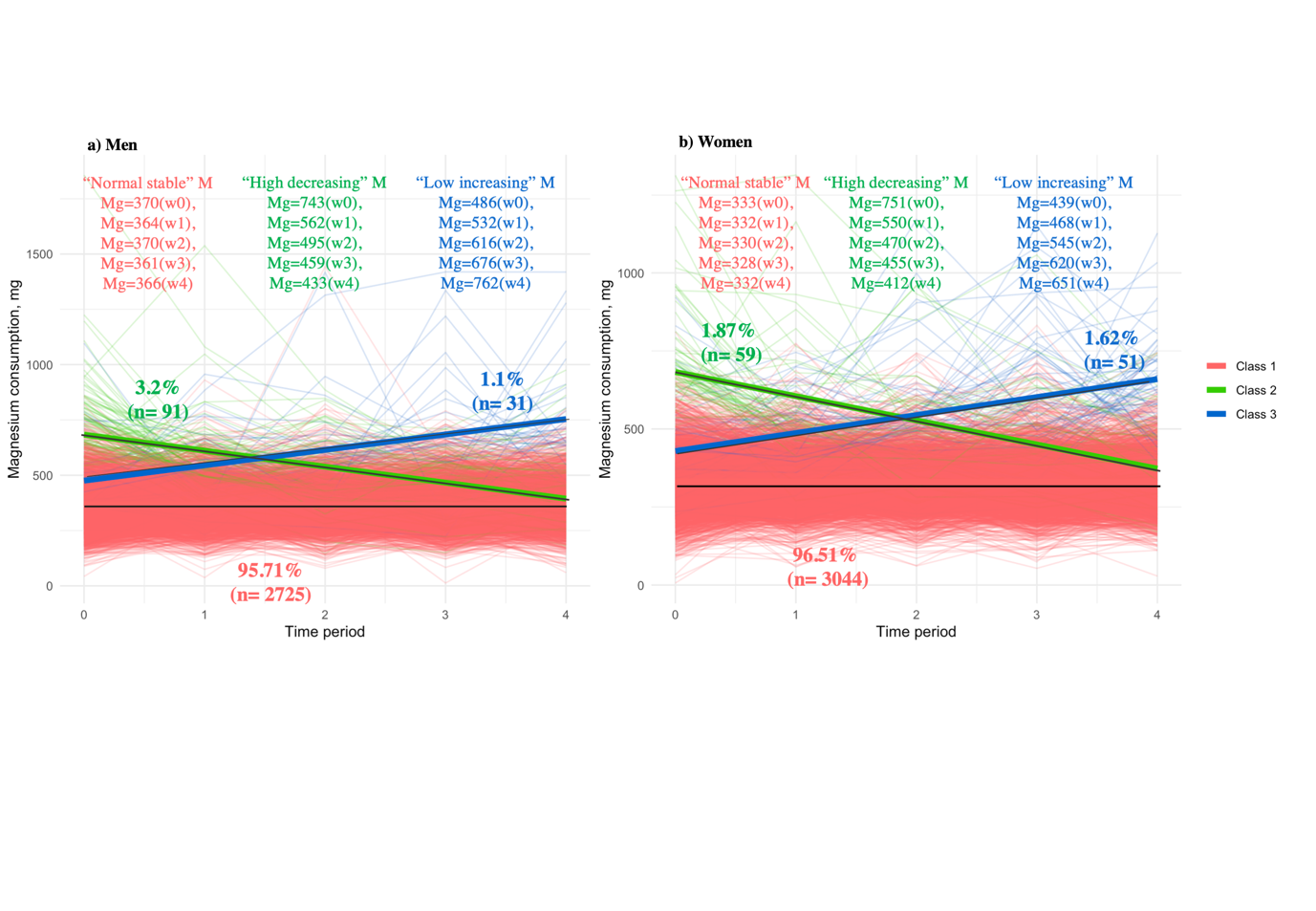

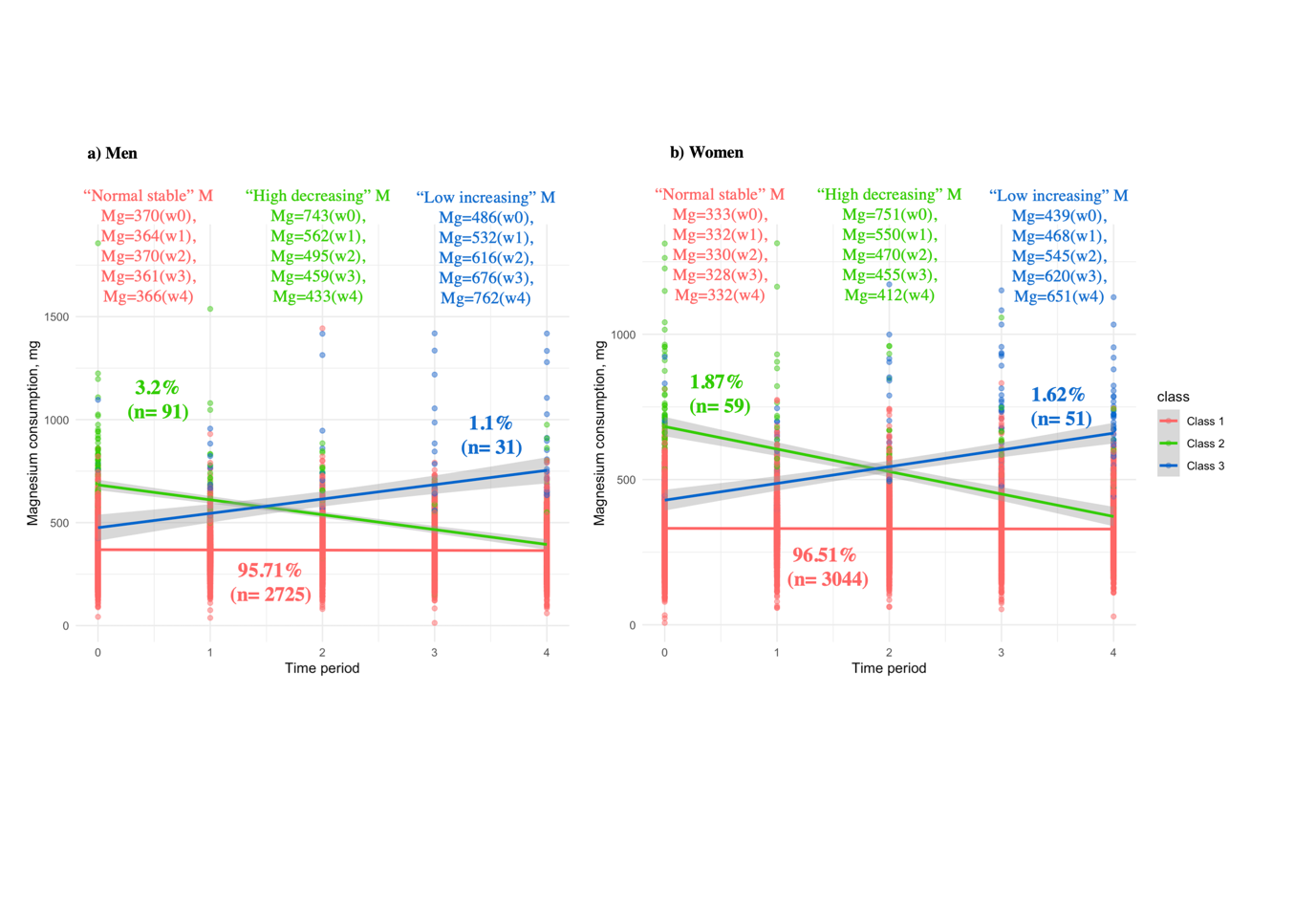
**

**Figure S.** Magnesium intake trajectories in women corresponding to different subgroups of individuals identified by latent class analysis. Note: “Time” is number of waves across 16 months.

**Table S2.** Intra-class correlation coefficient (ICC) between Magnesium intake classes in women corresponding to different subgroups of individuals identified by latent class analysis. Notes. A linear mixed effects model was estimated with random intercept and slope that vary between classes.

|  | **Magnesium (men)** | | | **Magnesium (women)** | | |
| --- | --- | --- | --- | --- | --- | --- |
| *Predictors* | *Estimates* | *CI* | *p* | *Estimates* | *CI* | *p* |
| (Intercept) | 508.99 | 327.80 – 690.17 | **<0.001** | 481.11 | 276.46 – 685.76 | **<0.001** |
| time | -1.32 | -81.50 – 78.86 | 0.974 | -6.79 | -83.36 – 69.78 | 0.862 |
| **Random Effects** | | | | | | |
| σ^2^ | 10040.37 | | | 7567.06 | | |
| τ_00_ | 25549.44 _class_ | | | 32651.35 _class_ | | |
| τ_11_ | 5006.15 _class.time_ | | | 4569.17 _class.time_ | | |
| ρ_01_ | -0.65 _class_ | | | -0.75 _class_ | | |
| ICC | 0.72 | | | 0.75 | | |
| N | 3 _class_ | | | 3 _class_ | | |
| Observations | 14235 | | | 15770 | | |
| Marginal R^2^ / Conditional R^2^ | 0.000 / 0.723 | | | 0.003 / 0.755 | | |

**Table S3. Comparison of latent classes by baseline characteristics in men participants**

| **Characteristic** | **Latent Class** | | | **p-values** | | |
| --- | --- | --- | --- | --- | --- | --- |
|  | **Class 1^1^** | **Class 2^1^** | **Class 3^1^** | **1 vs. 2^2^** | **2 vs. 3^2^** | **1 vs. 3^2^** |
| Age, year (SD) | 56 (8) | 56 (7) | 53 (8) | 0.9 | 0.071 | 0.043 |
| SBP Wave 1, mmHg (SD) | 138 (17) | 137 (16) | 136 (16) | 0.3 | >0.9 | 0.5 |
| SBP Wave 2, mmHg (SD) | 140 (15) | 139 (15) | 135 (14) | 0.8 | 0.2 | 0.082 |
| DBP Wave 1, mmHg (SD) | 83 (10) | 83 (11) | 82 (10) | 0.8 | 0.5 | 0.3 |
| DBP Wave 2, mmHg (SD) | 80 (9) | 80 (9) | 78 (9) | >0.9 | 0.2 | 0.069 |
| MAP Wave 1, mmHg (SD) | 102 (11) | 101 (12) | 100 (12) | 0.6 | 0.6 | 0.4 |
| MAP Wave 2, mmHg (SD) | 100 (11) | 100 (12) | 97 (12) | 0.9 | 0.2 | 0.086 |
| PP Wave 1, mmHg (SD) | 55 (11) | 54 (11) | 54 (11) | 0.2 | 0.8 | 0.7 |
| PP Wave 2, mmHg (SD) | 60 (13) | 60 (11) | 58 (11) | 0.6 | 0.2 | 0.4 |
| Education level, n (%) |  |  |  | 0.2 | 0.2 | 0.14 |
| Higher education | 1,407 (52%) | 57 (63%) | 13 (42%) |  |  |  |
| Diploma | 210 (7.7%) | 3 (3.3%) | 0 (0%) |  |  |  |
| High school | 428 (16%) | 11 (12%) | 8 (26%) |  |  |  |
| Secondary school | 586 (22%) | 16 (18%) | 8 (26%) |  |  |  |
| Other professional qualifications | 94 (3.4%) | 4 (4.4%) | 2 (6.5%) |  |  |  |
| Antihypertensive medication, n (%) | 225 (8.3%) | 10 (11%) | 2 (6.5%) | 0.3 | 0.7 | >0.9 |
| HDL mmol/L, (SD) | 1.32 (0.29) | 1.33 (0.27) | 1.37 (0.36) | >0.9 | 0.7 | 0.6 |
| Cholesterol, mmol/L, (SD) | 5.63 (1.07) | 5.39 (0.98) | 5.41 (1.08) | 0.062 | >0.9 | 0.3 |
| BMI, kg/m2 (SD) | 27.1 (3.7) | 27.3 (4.4) | 26.2 (3.4) | 0.8 | 0.3 | 0.2 |
| Diabetes mellitus, n (%) | 96 (3.5%) | 3 (3.3%) | 0 (0%) | >0.9 | 0.6 | 0.6 |
| Hypertension, n (%) | 1,293 (48%) | 49 (54%) | 13 (43%) | 0.2 | 0.4 | 0.7 |
| log White matter lesions | 7.50 (0.66) | 7.62 (0.63) | 7.46 (0.59) | 0.040 | 0.3 | >0.9 |
| Gray matter volume, mm3 (SD) | 702,215 (53,190) | 703,755 (60,835) | 733,295 (51,477) | 0.7 | 0.012 | <0.001 |
| Left hippocampal volume, mm3 (SD) | 3,827 (400) | 3,815 (427) | 3,950 (398) | 0.9 | 0.2 | 0.2 |
| Right hippocampal volume, mm3 (SD) | 3,949 (409) | 3,958 (419) | 4,094 (404) | 0.9 | 0.2 | 0.074 |
| Intracranial volume, mm3 (SD) | 1,642,509 (133,563) | 1,652,444 (143,547) | 1,719,519 (117,186) | 0.4 | 0.024 | 0.001 |
| Magnesium Wave 1, mg (SD) | 370 (110) | 743 (195) | 486 (178) | <0.001 | <0.001 | <0.001 |
| Magnesium Wave 2, mg (SD) | 364 (90) | 562 (168) | 532 (181) | <0.001 | 0.4 | <0.001 |
| Magnesium Wave 3, mg (SD) | 370 (91) | 495 (139) | 616 (246) | <0.001 | 0.003 | <0.001 |
| Magnesium Wave 4, mg (SD) | 361 (91) | 459 (114) | 676 (288) | <0.001 | <0.001 | <0.001 |
| Magnesium Wave 5, mg (SD) | 366 (92) | 433 (146) | 762 (249) | <0.001 | <0.001 | <0.001 |
| Ever Smoked, n (%) | 1,576 (58%) | 45 (49%) | 20 (65%) | 0.12 | 0.2 | 0.6 |
| Higher education, n (%) | 1,407 (52%) | 57 (63%) | 13 (42%) | 0.045 | 0.063 | 0.4 |
| Alcohol intake n (%) | 1,938 (71%) | 60 (66%) | 22 (71%) | 0.3 | 0.6 | >0.9 |
| *^1^* Mean (SD); n (%)  *^2^* Wilcoxon rank sum test; Fisher's Exact Test for Count Data with simulated p-value (based on 2000 replicates) | | | | | | |

**Table S4. Comparison of latent classes by baseline characteristics in women participants**

| **Characteristic** | **Latent Class** | | | **p-values** | | |
| --- | --- | --- | --- | --- | --- | --- |
|  | **Class 1^1^** | **Class 2^1^** | **Class 3^1^** | **1 vs. 2^2^** | **2 vs. 3^2^** | **1 vs. 3^2^** |
| Age, year (SD) | 55 (7) | 55 (7) | 55 (7) | 0.8 | 0.5 | 0.5 |
| SBP Wave 1, mmHg (SD) | 132 (18) | 132 (22) | 135 (22) | 0.9 | 0.5 | 0.3 |
| SBP Wave 2, mmHg (SD) | 133 (16) | 135 (17) | 135 (18) | 0.5 | 0.8 | 0.3 |
| DBP Wave 1, mmHg (SD) | 79 (10) | 80 (11) | 80 (13) | >0.9 | >0.9 | >0.9 |
| DBP Wave 2, mmHg (SD) | 77 (9) | 76 (10) | 77 (11) | 0.9 | 0.6 | 0.5 |
| MAP Wave 1, mmHg (SD) | 97 (12) | 97 (14) | 98 (15) | >0.9 | 0.8 | 0.6 |
| MAP Wave 2, mmHg (SD) | 95 (11) | 96 (13) | 96 (14) | 0.6 | >0.9 | 0.5 |
| PP Wave 1, mmHg (SD) | 52 (13) | 53 (14) | 55 (14) | 0.7 | 0.5 | 0.2 |
| PP Wave 2, mmHg (SD) | 57 (14) | 60 (15) | 56 (11) | 0.075 | 0.2 | >0.9 |
| Education level, n (%) |  |  |  | 0.6 | 0.8 | 0.060 |
| Higher education | 1,414 (46%) | 33 (56%) | 33 (65%) |  |  |  |
| Diploma | 95 (3.1%) | 1 (1.7%) | 0 (0%) |  |  |  |
| High school | 546 (18%) | 8 (14%) | 5 (9.8%) |  |  |  |
| Secondary school | 822 (27%) | 13 (22%) | 9 (18%) |  |  |  |
| Other professional qualifications | 167 (5.5%) | 4 (6.8%) | 4 (7.8%) |  |  |  |
| Antihypertensive medication, n (%) | 200 (6.6%) | 3 (5.1%) | 4 (7.8%) | >0.9 | 0.7 | 0.6 |
| HDL mmol/L, (SD) | 1.64 (0.36) | 1.64 (0.34) | 1.67 (0.43) | >0.9 | >0.9 | 0.9 |
| Cholesterol, mmol/L, (SD) | 5.86 (1.06) | 5.66 (1.08) | 5.93 (1.01) | 0.11 | 0.14 | 0.7 |
| BMI, kg/m2 (SD) | 26.0 (4.4) | 25.6 (5.7) | 25.0 (3.8) | 0.10 | >0.9 | 0.12 |
| Diabetes mellitus, n (%) | 67 (2.2%) | 1 (1.7%) | 0 (0%) | >0.9 | >0.9 | 0.6 |
| Hypertension, n (%) | 1,022 (34%) | 20 (34%) | 21 (41%) | >0.9 | 0.6 | 0.3 |
| log White matter lesions | 7.26 (0.64) | 7.18 (0.59) | 7.36 (0.71) | 0.5 | 0.3 | 0.3 |
| Gray matter volume, mm3 (SD) | 638,041 (46,423) | 631,252 (55,549) | 637,273 (44,027) | 0.2 | 0.4 | >0.9 |
| Left hippocampal volume, mm3 (SD) | 3,576 (351) | 3,556 (342) | 3,511 (328) | 0.7 | 0.5 | 0.2 |
| Right hippocampal volume, mm3 (SD) | 3,685 (352) | 3,694 (340) | 3,628 (367) | 0.9 | 0.4 | 0.4 |
| Intracranial volume, mm3 (SD) | 1,473,337 (116,307) | 1,439,306 (130,266) | 1,490,808 (112,418) | 0.048 | 0.049 | 0.4 |
| Magnesium Wave 1, mg (SD) | 333 (97) | 751 (181) | 439 (148) | <0.001 | <0.001 | <0.001 |
| Magnesium Wave 2, mg (SD) | 332 (79) | 550 (189) | 468 (124) | <0.001 | 0.022 | <0.001 |
| Magnesium Wave 3, mg (SD) | 330 (77) | 470 (163) | 545 (182) | <0.001 | 0.012 | <0.001 |
| Magnesium Wave 4, mg (SD) | 328 (81) | 455 (140) | 620 (214) | <0.001 | <0.001 | <0.001 |
| Magnesium Wave 5, mg (SD) | 332 (77) | 412 (124) | 651 (157) | <0.001 | <0.001 | <0.001 |
| Smoking status, n (%) | 1,998 (66%) | 45 (76%) | 27 (53%) | 0.11 | 0.018 | 0.077 |
| Higher education, n (%) | 1,414 (46%) | 33 (56%) | 33 (65%) | 0.2 | 0.4 | 0.008 |
| Alcohol intake n (%) | 2,473 (81%) | 52 (88%) | 43 (84%) | 0.2 | 0.6 | 0.7 |
| *^1^* Mean (SD); n (%)  *^2^* Wilcoxon rank sum test; Fisher's Exact Test for Count Data with simulated p-value (based on 2000 replicates) | | | | | | |

**Table S5. Model 1 of the association between Mg intake trajectories over time and brain volumes at UK biobank study**

|  | **Gray matter volume (mm^3^)** | | **White matter volume (mm^3^)** | | **Left hippocampal volume (mm^3^)** | | **Right hippocampal volume (mm^3^)** | | **White matter lesions (mm^3^)** | |
| --- | --- | --- | --- | --- | --- | --- | --- | --- | --- | --- |
|  | **Men** | **Women** | **Men** | **Women** | **Men** | **Women** | **Men** | **Women** | **Men** | **Women** |
|  | **B (SE)** | **B (SE)** | **B (SE)** | **B (SE)** | **B (SE)** | **B (SE)** | **B (SE)** | **B (SE)** | **B (SE)** | **B (SE)** |
| High decreasing | -1,451.746 (1,470.698) | 3,592.797^**^ (1,544.504) | -1,008.177 (1,495.768) | 5,392.171^****^ (1,567.827) | -24.282 (15.529) | 23.488 (16.942) | -2.593 (15.914) | 56.800^****^ (16.912) | 0.103^****^ (0.026) | -0.021 (0.032) |
| low increasing | 2,094.169 (2,497.932) | -5,655.619^****^ (1,658.566) | 4,478.254^*^ (2,540.512) | -4,434.954^***^ (1,683.611) | -35.336 (26.376) | -81.163^****^ (18.193) | -16.077 (27.029) | -75.704^****^ (18.160) | -0.032 (0.044) | 0.054 (0.034) |
| Constant | 334,028.700^****^ (3,850.372) | 241,674.200^****^ (3,260.513) | 92,408.360^****^ (3,916.005) | 29,296.480^****^ (3,309.750) | 2,835.771^****^ (40.657) | 2,320.907^****^ (35.766) | 2,755.018^****^ (41.663) | 2,193.567^****^ (35.701) | 2.717^****^ (0.068) | 2.895^****^ (0.067) |
| Observations | 14,235 | 15,770 | 14,235 | 15,770 | 14,235 | 15,770 | 14,235 | 15,770 | 14,235 | 15,770 |
| R^2^ | 0.668 | 0.682 | 0.663 | 0.656 | 0.342 | 0.324 | 0.337 | 0.334 | 0.310 | 0.295 |
| Adjusted R^2^ | 0.668 | 0.682 | 0.663 | 0.656 | 0.341 | 0.324 | 0.337 | 0.334 | 0.310 | 0.295 |
| Residual Std. Error | 30,842.820 (df = 14229) | 26,248.460 (df = 15764) | 31,368.560 (df = 14229) | 26,644.840 (df = 15764) | 325.674 (df = 14229) | 287.929 (df = 15764) | 333.735 (df = 14229) | 287.407 (df = 15764) | 0.544 (df = 14229) | 0.535 (df = 15764) |
| F Statistic | 5,720.499^****^ (df = 5; 14229) | 6,769.925^****^ (df = 5; 15764) | 5,591.815^****^ (df = 5; 14229) | 6,020.109^****^ (df = 5; 15764) | 1,476.861^****^ (df = 5; 14229) | 1,510.379^****^ (df = 5; 15764) | 1,445.392^****^ (df = 5; 14229) | 1,582.841^****^ (df = 5; 15764) | 1,281.520^****^ (df = 5; 14229) | 1,317.837^****^ (df = 5; 15764) |
| Note: | .* p<0.05; ** p<0.01; *** p<0.001 | | | | | | | | | |

Note: Abbreviations: SE, standard error; GMV, gray matter, LHCV; left hippocampus, RHCV; right hippocampus, WMLs; white matter lesions, and ICV; intracranial volume. Hierarchical regression analysis results of the association between Mg intake classes (High decreasing vs normal stable and low increasing vs normal stable) and the brain volumes including GMV, LHCV, RHCV, and WMLs, at UK biobank study. Model 1 was **adjusted** for the main covariates including age, education and **ICV**. Data represents unstandardized Beta correlation +/- SE (p). Beta is per one mg unit increment in Mg intake variables and expressed in SD units of the dependent variable.

**Table S6 Model 2 of the association between Mg intake trajectories over time and brain volumes at UK biobank study**

|  | **Gray matter volume (mm^3^)** | | **White matter volume (mm^3^)** | | **Left hippocampal volume (mm^3^)** | | **Right hippocampal volume (mm^3^)** | | **White matter lesions (mm^3^)** | |
| --- | --- | --- | --- | --- | --- | --- | --- | --- | --- | --- |
|  | **Men** | **Women** | **Men** | **Women** | **Men** | **Women** | **Men** | **Women** | **Men** | **Women** |
|  | **B (SE)** | **B (SE)** | **B (SE)** | **B (SE)** | **B (SE)** | **B (SE)** | **B (SE)** | **B (SE)** | **B (SE)** | **B (SE)** |
| High decreasing | -4,692.328 (3,274.945) | 8,727.089^**^ (3,745.013) | -9,975.175^***^ (3,339.036) | 3,871.576 (3,804.020) | -33.616 (34.661) | 36.510 (41.122) | 2.513 (35.518) | 108.526^***^ (41.031) | 0.024 (0.058) | -0.130^*^ (0.076) |
| low increasing | 4,931.994 (3,149.386) | -10,069.670^****^ (1,931.433) | 5,473.484^*^ (3,211.019) | -2,979.127 (1,961.865) | 46.937 (33.332) | -87.404^****^ (21.208) | 33.403 (34.156) | -55.530^***^ (21.161) | -0.045 (0.055) | 0.097^**^ (0.039) |
| Baseline Mg | 8.581^****^ (2.417) | 6.472^***^ (2.199) | -3.572 (2.465) | 3.674 (2.234) | 0.022 (0.026) | 0.066^***^ (0.024) | 0.085^***^ (0.026) | 0.090^****^ (0.024) | -0.0001^***^ (0.00004) | 0.0001^**^ (0.00004) |
| Baseline Mg x High decreasing | 0.660 (7.828) | -19.654^**^ (8.781) | 26.272^****^ (7.981) | -0.195 (8.919) | 0.004 (0.083) | -0.102 (0.096) | -0.092 (0.085) | -0.223^**^ (0.096) | 0.0003^**^ (0.0001) | 0.0002 (0.0002) |
| Baseline Mg x low increasing | -28.706^**^ (14.325) | 42.725^****^ (11.429) | -4.456 (14.606) | -20.268^*^ (11.609) | -0.623^****^ (0.152) | -0.005 (0.125) | -0.436^***^ (0.155) | -0.331^***^ (0.125) | 0.0002 (0.0003) | -0.001^***^ (0.0002) |
| Constant | 332,818.000^****^ (3,852.407) | 242,378.800^****^ (3,267.690) | 91,610.470^****^ (3,927.798) | 29,689.490^****^ (3,319.176) | 2,833.363^****^ (40.772) | 2,328.727^****^ (35.881) | 2,757.318^****^ (41.781) | 2,204.519^****^ (35.802) | 2.753^****^ (0.068) | 2.912^****^ (0.066) |
| Observations | 14,235 | 15,770 | 14,235 | 15,770 | 14,235 | 15,770 | 14,235 | 15,770 | 14,235 | 15,770 |
| R^2^ | 0.670 | 0.683 | 0.663 | 0.657 | 0.343 | 0.324 | 0.338 | 0.335 | 0.318 | 0.302 |
| Adjusted R^2^ | 0.670 | 0.683 | 0.663 | 0.657 | 0.342 | 0.324 | 0.337 | 0.335 | 0.317 | 0.301 |
| Residual Std. Error | 30,755.590 (df = 14225) | 26,217.580 (df = 15760) | 31,357.470 (df = 14225) | 26,630.670 (df = 15760) | 325.503 (df = 14225) | 287.883 (df = 15760) | 333.557 (df = 14225) | 287.247 (df = 15760) | 0.541 (df = 14225) | 0.533 (df = 15760) |
| F Statistic | 3,205.532^****^ (df = 9; 14225) | 3,774.508^****^ (df = 9; 15760) | 3,110.324^****^ (df = 9; 14225) | 3,350.373^****^ (df = 9; 15760) | 823.441^****^ (df = 9; 14225) | 840.360^****^ (df = 9; 15760) | 805.998^****^ (df = 9; 14225) | 882.726^****^ (df = 9; 15760) | 735.496^****^ (df = 9; 14225) | 757.201^****^ (df = 9; 15760) |
|  | .* p<0.05; ** p<0.01; *** p<0.001 | | | | | | | | | |

Note: Abbreviations: SE, standard error; GMV, gray matter, LHCV; left hippocampus, RHCV; right hippocampus, WMLs; white matter lesions, and ICV; intracranial volume. Hierarchical regression analysis results of the association between Mg intake classes (High decreasing vs normal stable and low increasing vs normal stable) and the brain volumes including GMV, LHCV, RHCV, and WMLs, at UK biobank study. Model 1 was adjusted for the main covariates including age, education and ICV. Model 2 was additionally tested the two-way interactions between baseline Mg x Mg classes while controlling for baseline Mg and antihypertensive medication. Data represents unstandardized Beta correlation +/- SE (p). Beta is per one mg unit increment in Mg intake variables and expressed in SD units of the dependent variable.

**Table S7 Model 2 of the association between Mg intake trajectories over time and brain volumes at UK biobank study**

|  | **Gray matter volume (mm^3^)** | | **White matter volume (mm^3^)** | | **Left hippocampal volume (mm^3^)** | | **Right hippocampal volume (mm^3^)** | | **White matter lesions (mm^3^)** | |
| --- | --- | --- | --- | --- | --- | --- | --- | --- | --- | --- |
|  | **Men** | **Women** | **Men** | **Women** | **Men** | **Women** | **Men** | **Women** | **Men** | **Women** |
|  | **B (SE)** | **B (SE)** | **B (SE)** | **B (SE)** | **B (SE)** | **B (SE)** | **B (SE)** | **B (SE)** | **B (SE)** | **B (SE)** |
| High decreasing | -3,845.243 (3,256.911) | 7,521.830^**^ (3,722.368) | -9,284.898^***^ (3,319.663) | 2,185.547 (3,770.945) | -28.579 (34.672) | 29.757 (40.965) | 8.319 (35.498) | 103.124^**^ (40.895) | 0.009 (0.057) | -0.113 (0.076) |
| low increasing | 3,874.717 (3,127.947) | -10,706.060^****^ (1,922.555) | 4,950.949 (3,188.214) | -3,945.020^**^ (1,947.644) | 42.825 (33.299) | -87.213^****^ (21.158) | 25.388 (34.092) | -55.489^***^ (21.122) | -0.028 (0.055) | 0.099^**^ (0.039) |
| Baseline Mg | 8.308^****^ (2.420) | 6.875^***^ (2.196) | -5.286^**^ (2.466) | 2.998 (2.225) | 0.032 (0.026) | 0.065^***^ (0.024) | 0.092^****^ (0.026) | 0.086^****^ (0.024) | -0.0001^***^ (0.00004) | 0.0001^**^ (0.00004) |
| Baseline Mg x High decreasing | 1.120 (7.787) | -16.802^*^ (8.732) | 26.491^****^ (7.937) | 4.557 (8.846) | -0.006 (0.083) | -0.086 (0.096) | -0.102 (0.085) | -0.209^**^ (0.096) | 0.0003^**^ (0.0001) | 0.0001 (0.0002) |
| Baseline Mg x low increasing | -25.613^*^ (14.229) | 43.123^****^ (11.365) | -9.988 (14.503) | -18.424 (11.513) | -0.602^****^ (0.151) | 0.005 (0.125) | -0.392^**^ (0.155) | -0.316^**^ (0.125) | 0.0002 (0.0003) | -0.001^**^ (0.0002) |
| Constant | 340,933.300^****^ (4,794.676) | 243,385.100^****^ (3,723.059) | 108,298.000^****^ (4,887.057) | 39,506.150^****^ (3,771.645) | 2,828.207^****^ (51.042) | 2,417.771^****^ (40.973) | 2,807.002^****^ (52.258) | 2,303.468^****^ (40.903) | 2.463^****^ (0.085) | 2.914^****^ (0.076) |
| Observations | 14,235 | 15,770 | 14,235 | 15,770 | 14,235 | 15,770 | 14,235 | 15,770 | 14,235 | 15,770 |
| R^2^ | 0.676 | 0.688 | 0.669 | 0.663 | 0.347 | 0.331 | 0.343 | 0.341 | 0.326 | 0.307 |
| Adjusted R^2^ | 0.675 | 0.687 | 0.669 | 0.663 | 0.346 | 0.330 | 0.342 | 0.340 | 0.325 | 0.306 |
| Residual Std. Error | 30,484.930 (df = 14218) | 26,037.990 (df = 15753) | 31,072.290 (df = 14218) | 26,377.790 (df = 15753) | 324.531 (df = 14218) | 286.550 (df = 15753) | 332.260 (df = 14218) | 286.063 (df = 15753) | 0.538 (df = 14218) | 0.531 (df = 15753) |
| F Statistic | 1,851.567^****^ (df = 16; 14218) | 2,166.622^****^ (df = 16; 15753) | 1,798.652^****^ (df = 16; 14218) | 1,940.307^****^ (df = 16; 15753) | 471.738^****^ (df = 16; 14218) | 486.734^****^ (df = 16; 15753) | 464.310^****^ (df = 16; 14218) | 509.262^****^ (df = 16; 15753) | 429.734^****^ (df = 16; 14218) | 435.739^****^ (df = 16; 15753) |
| *Note:* | .* p<0.05; ** p<0.01; *** p<0.001 | | | | | | | | | |

Note: Abbreviations: SE, standard error; GMV, gray matter volume, WMV; white matter volume, LHCV; left hippocampus volume, RHCV; right hippocampus volume, WMLs; white matter lesions, and ICV; intracranial volume. Hierarchical regression analysis results of the association between Mg intake trajectories (“High decreasing” vs “normal stable” and “low increasing” vs” normal stable”) and the brain volumes including GMV, LHCV, RHCV, and WMLs, at UK biobank study. Model 1 was **adjusted** for the main covariates including age, education and **ICV**. Model 2 was additionally tested the two-way interactions between baseline Mg x Mg trajectories while controlling for **baseline** Mg **and** antihypertensive medication. **Model 3 was** additionally **adjusted for the other covariates: age, education and antihypertensive medication HDL, cholesterol, diabetes mellitus, smoking status, higher education, physical activity, and alcohol intake.** Data represents unstandardized Beta correlation +/- SE (p). Beta is per one mg unit increment in Mg intake variables and expressed in SD units of the dependent variable.

**
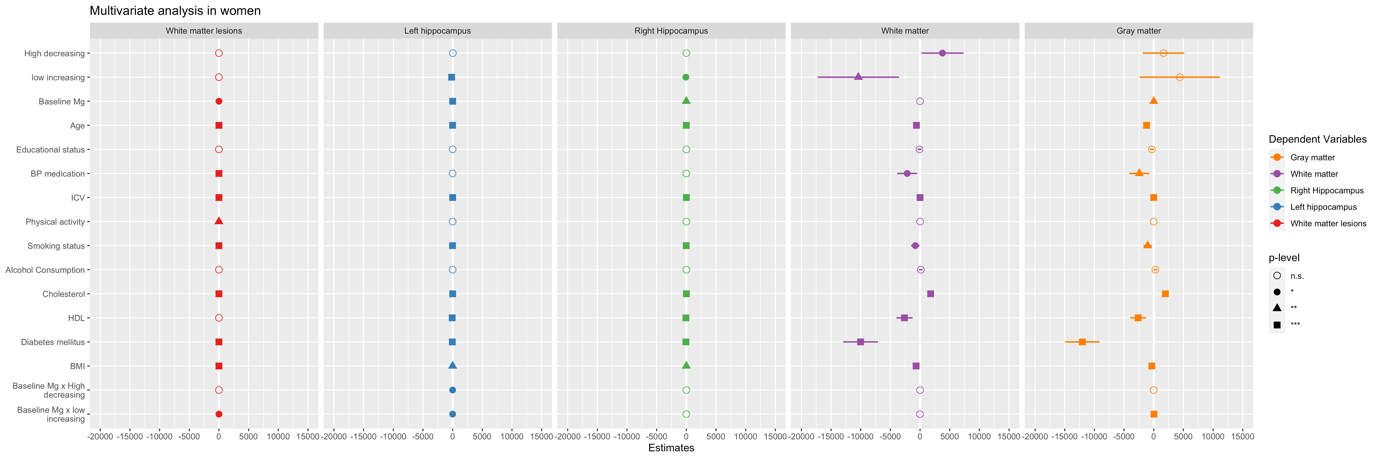

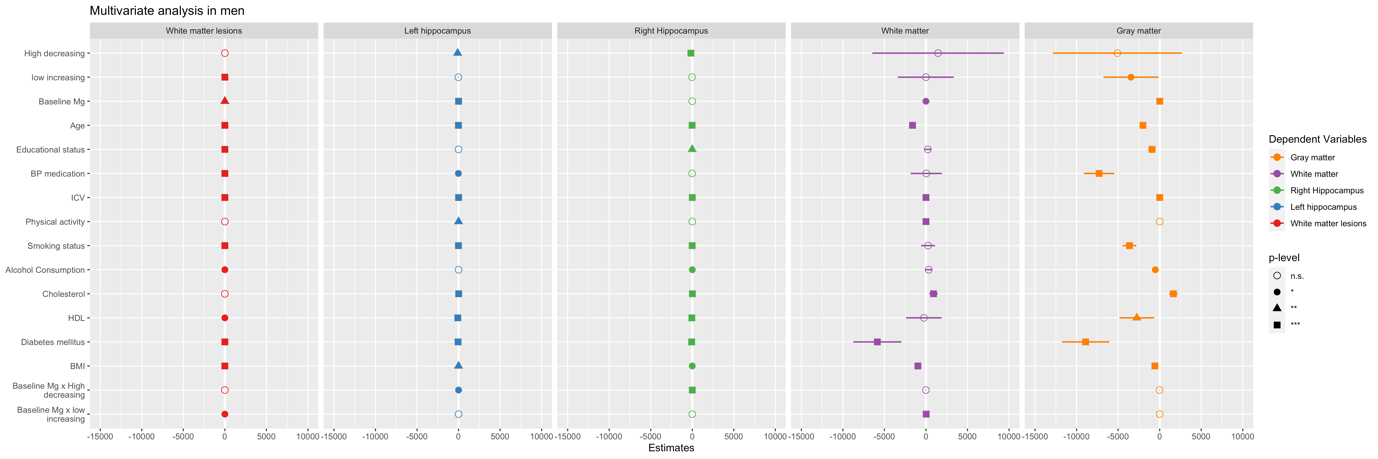
**

**Figure S6 Hierarchical regression analysis results of the association between Magnesium intake classes and brain volumes including gray matter, left hippocampal, right hippocampal, white matter lesions at UK biobank study.** **Model 3 was adjusted for Baseline Mg x classes, and main covariates: age, intracranial volume, HDL, cholesterol, diabetes, smoking status, higher education, physical activity, alcohol intake, and antihypertensive medication. Note. Error bars represent standard error from the same model.**


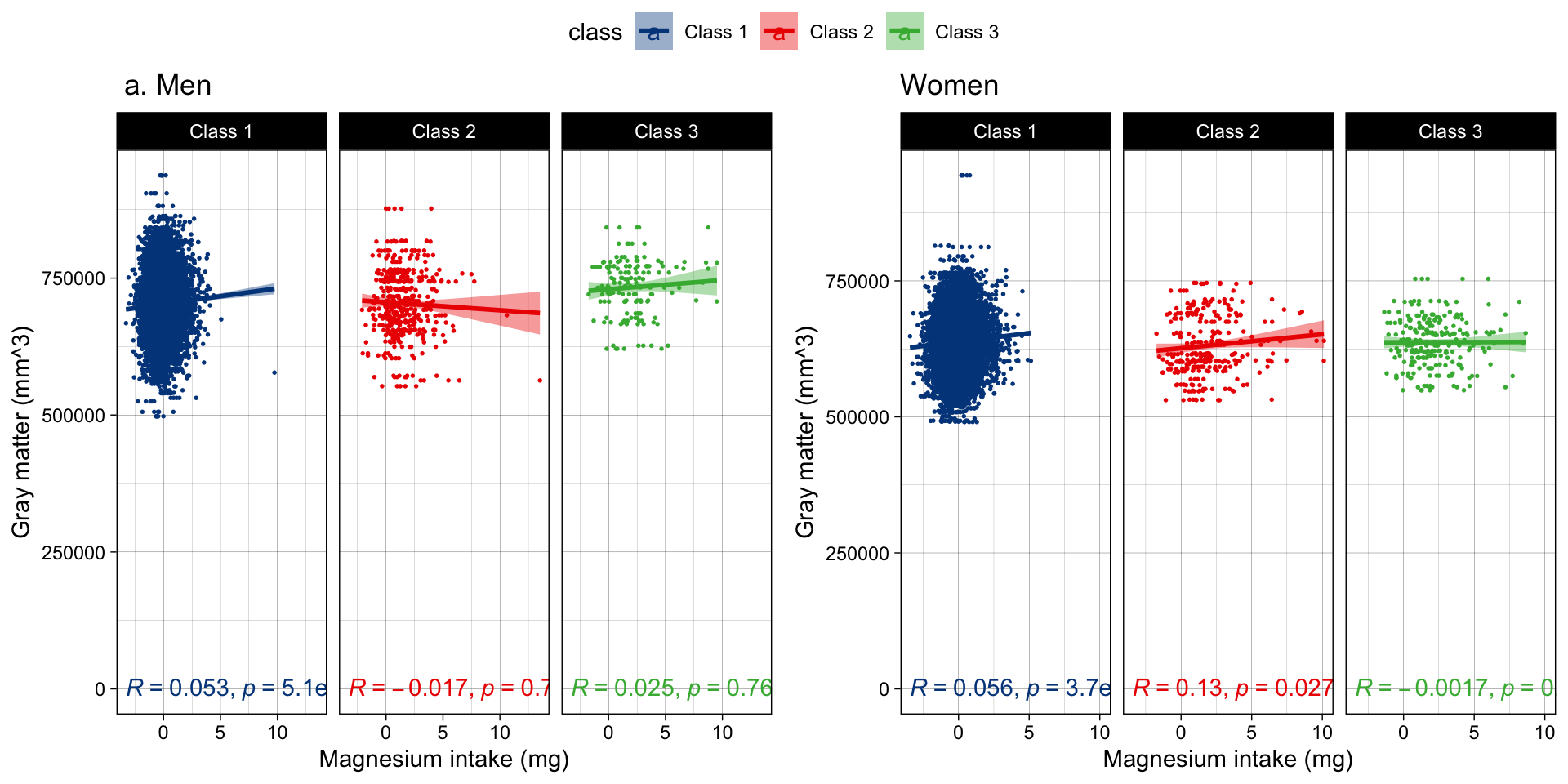


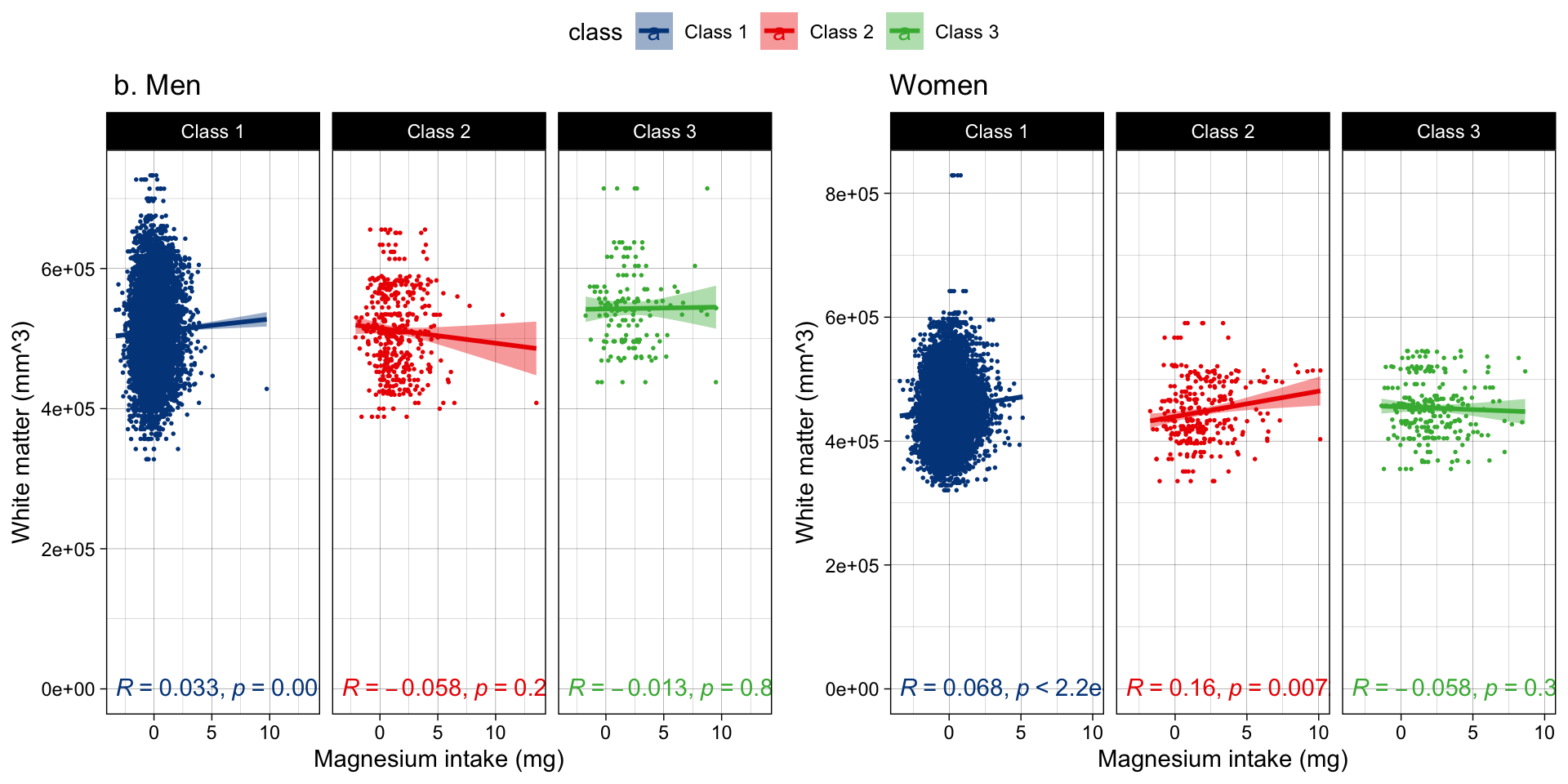


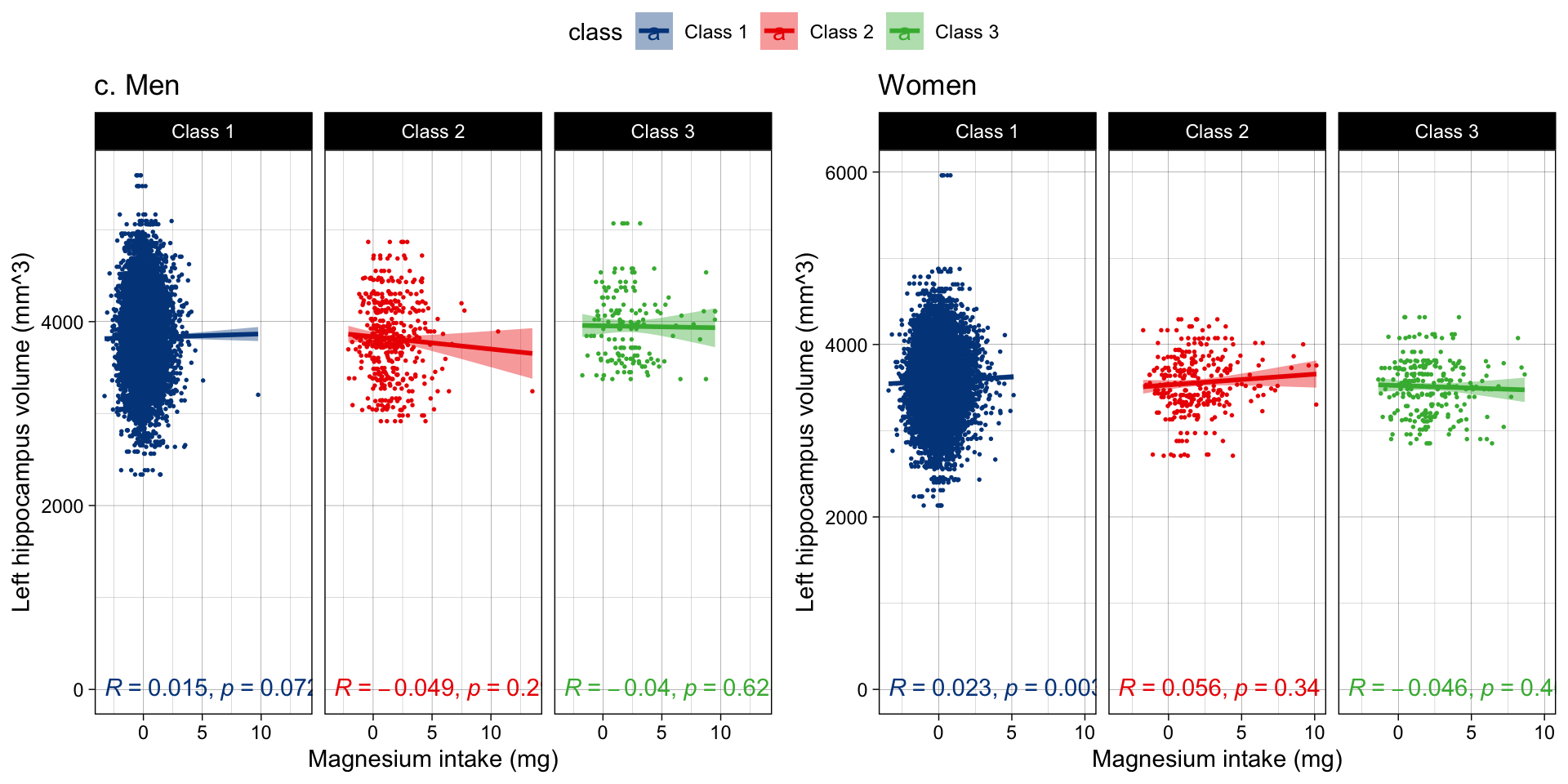


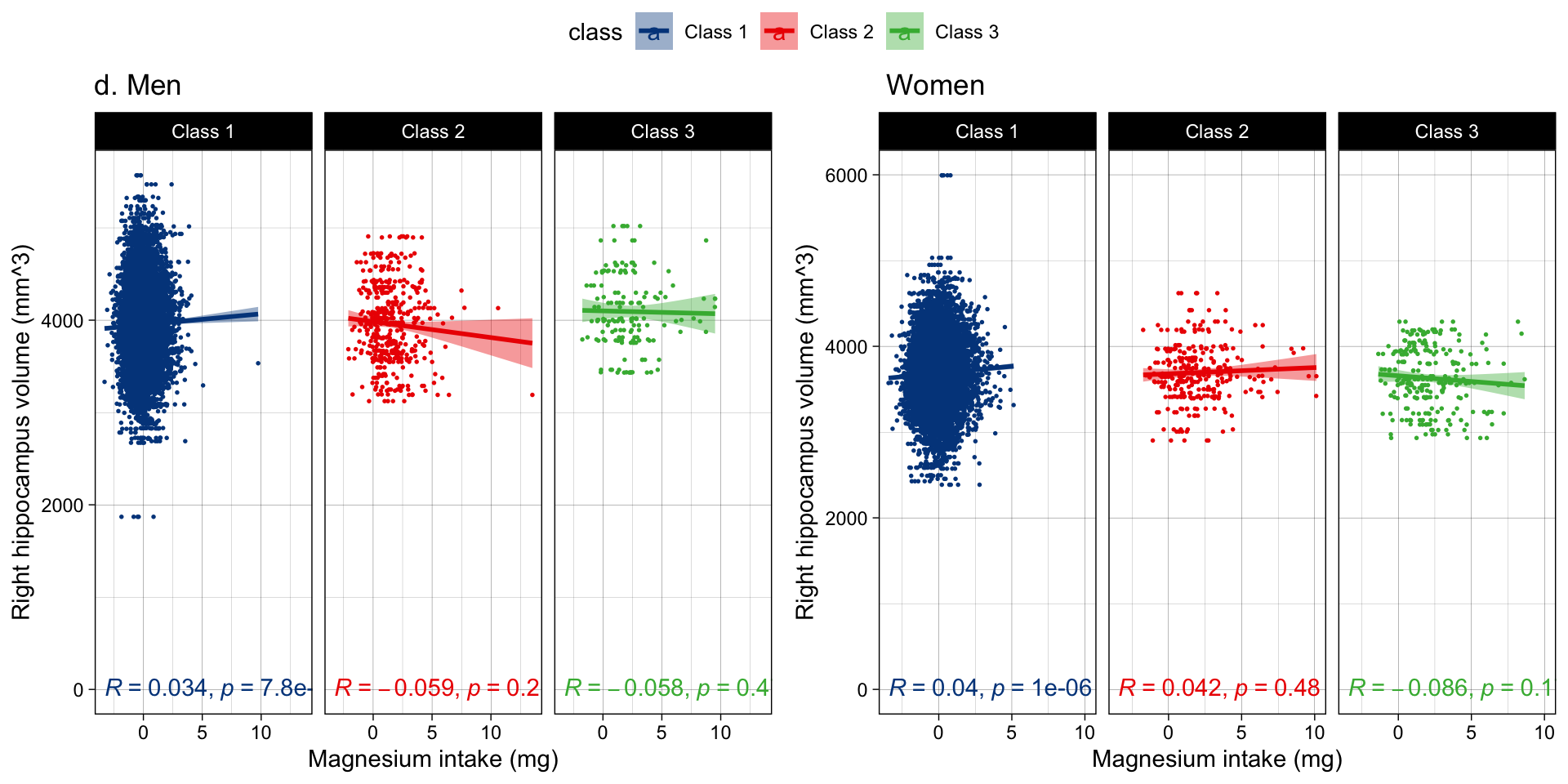


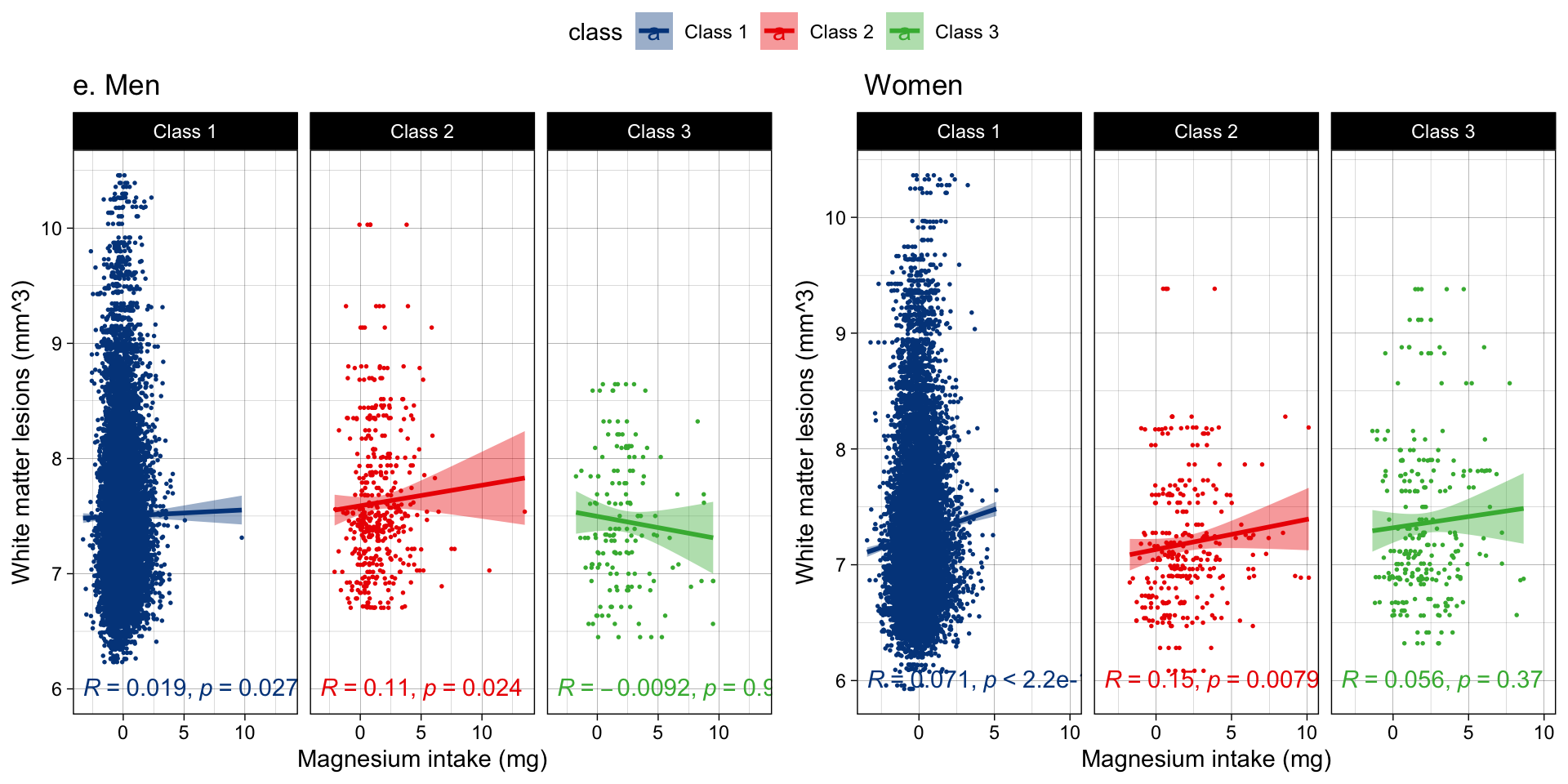


**Figure S7 the scatter plots show the association direction between Magnesium intake classes (class1 [normal stable], class 2 [high decreasing], class 3[low increasing]) and brain volumes including a) gray matter; b) white matter; c) left hippocampal, d) right hippocampal, e) white matter lesions in men and women at UK biobank study.** **Model 3 was assessed the two-way interaction between baseline Mg and Mg trajectories and adjusted for main covariates: age, intracranial volume, HDL, cholesterol, diabetes, smoking status, higher education, physical activity, alcohol intake, and antihypertensive medication.** **Significance is at a < 0.05.**


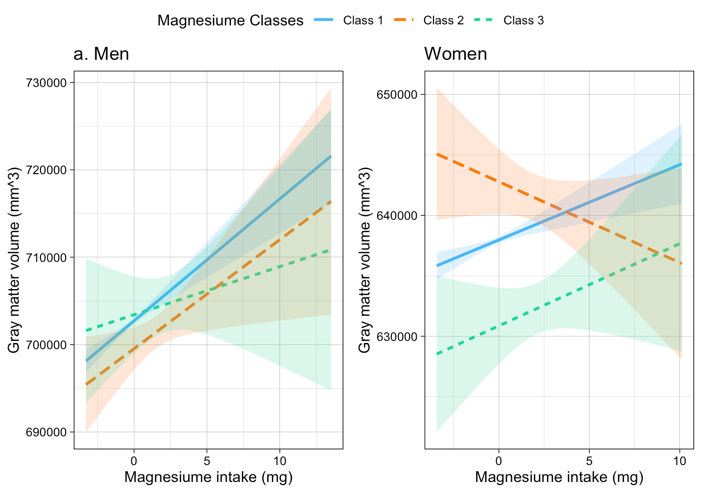

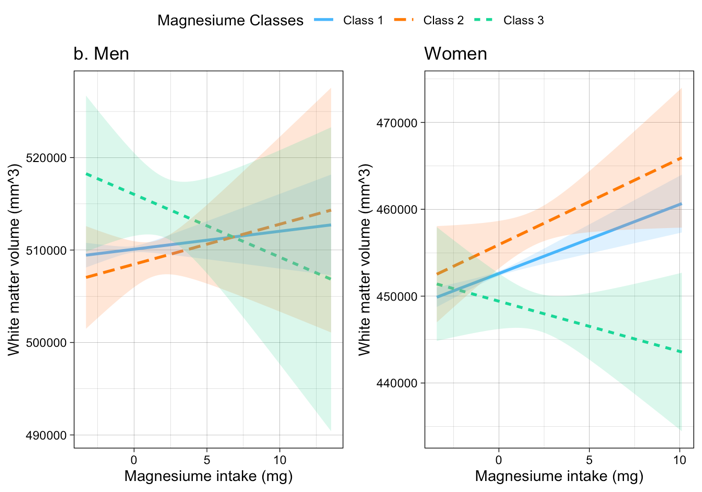

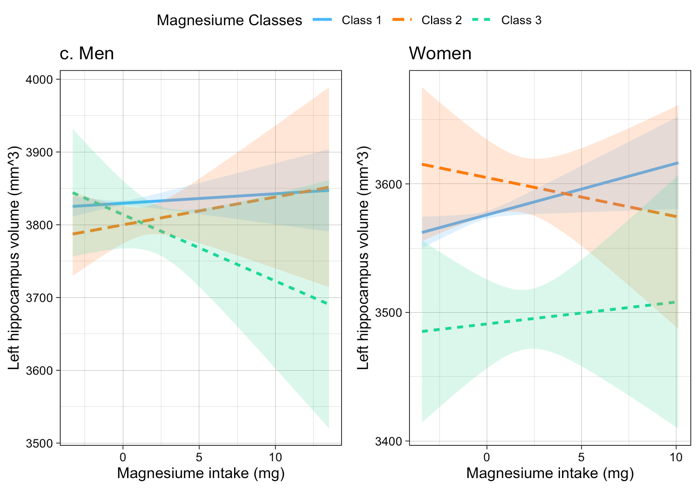

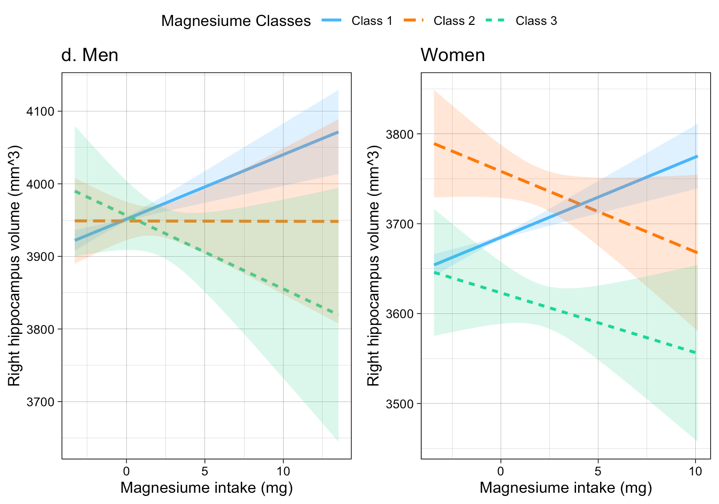

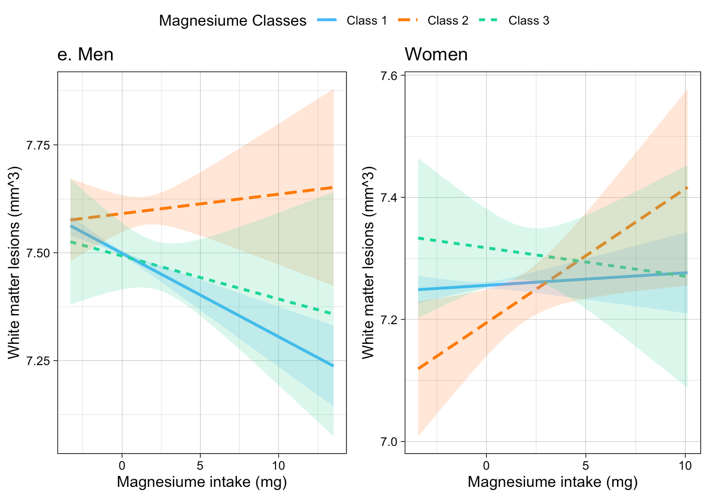


**Figure S8.** Two-way interaction between baseline Mg and Mg classes and brain volumes including a) gray matter; b) white matter; c) left hippocampal; d) right hippocampal; e) white matter lesions, volume in men and women.

**Table S8.** Model3 of associations between baseline magnesium intake and baseline BP.

|  | **MAP (mmHg)** | | | | **SBP (mmHg)** | | | | **DBP (mmHg)** | | | | **PP (mmHg)** | | | |
| --- | --- | --- | --- | --- | --- | --- | --- | --- | --- | --- | --- | --- | --- | --- | --- | --- |
|  | **Men** | | **Women** | | **Men** | | **Women** | | **Men** | | **Women** | | **Men** | | **Women** | |
|  | | **B (SE)** | | **B (SE)** | | **B (SE)** | | **B (SE)** | | **B (SE)** | | **B (SE)** | | **B (SE)** | | **B (SE)** |
| Baseline Mg | -0.001 (0.001) | | 0.0001 (0.001) | | -0.001 (0.001) | | 0.001 (0.001) | | -0.001 (0.001) | | -0.0005 (0.001) | | 0.0001 (0.001) | | 0.002^**^ (0.001) | |
| Constant | 90.172^****^ (0.695) | | 76.251^****^ (0.668) | | 106.978^****^ (0.995) | | 84.136^****^ (1.014) | | 81.768^****^ (0.613) | | 72.308^****^ (0.568) | | 25.210^****^ (0.662) | | 11.828^****^ (0.707) | |
|  | | | | | | | | | | | | | | | | |
| Observations | 14,235 | | 15,770 | | 14,235 | | 15,770 | | 14,235 | | 15,770 | | 14,235 | | 15,770 | |
| R^2^ | 0.052 | | 0.099 | | 0.093 | | 0.159 | | 0.030 | | 0.047 | | 0.136 | | 0.191 | |
| Adjusted R^2^ | 0.052 | | 0.098 | | 0.093 | | 0.158 | | 0.030 | | 0.046 | | 0.136 | | 0.191 | |
| Residual Std. Error | 11.027 (df = 14230) | | 11.113 (df = 15765) | | 15.776 (df = 14230) | | 16.852 (df = 15765) | | 9.727 (df = 14230) | | 9.448 (df = 15765) | | 10.488 (df = 14230) | | 11.750 (df = 15765) | |
| F Statistic | 195.185^****^ (df = 4; 14230) | | 430.988^****^ (df = 4; 15765) | | 366.558^****^ (df = 4; 14230) | | 742.823^****^ (df = 4; 15765) | | 111.205^****^ (df = 4; 14230) | | 192.676^****^ (df = 4; 15765) | | 560.063^****^ (df = 4; 14230) | | 930.938^****^ (df = 4; 15765) | |
|  | | | | | | | | | | | | | | | | |
| Note: | .* p<0.05; ** p<0.01; *** p<0.001 | | | | | | | | | | | | | | | |

**Abbreviations: SE, standard error; MAP, mean arterial pressure; SBP, systolic blood pressure; DBP, diastolic blood pressure.** Hierarchical regression analysis results of the association between baseline magnesium intake and baseline BP (MAP, SBP, DBP, PP) at UK biobank study. Model 1 was **adjusted** for the main covariates including age, education. Model 2 was additionally adjusted for antihypertensive medication. **Model 3 was** additionally **adjusted for other covariates: HDL, cholesterol, diabetes, smoking status, higher education, physical activity, and alcohol intake.** Data represents unstandardized Beta correlation +/- SE (p). Beta is per one mg unit increment in Magnesium intake variables and expressed in SD units of the dependent variable**.**

**Table S9 Model 1 of the association between Mg intake trajectories over time and blood pressure changes at UK biobank study**

|  | **Δ MAP (mmHg)** | | **Δ SBP (mmHg)** | | **Δ DBP (mmHg)** | | **Δ PP (mmHg)** | |
| --- | --- | --- | --- | --- | --- | --- | --- | --- |
|  | **Men** | **Women** | **Men** | **Women** | **Men** | **Women** | **Men** | **Women** |
|  | **B (SE)** | **B (SE)** | **B (SE)** | **B (SE)** | **B (SE)** | **B (SE)** | **B (SE)** | **B (SE)** |
| High decreasing pattern | -0.618 (0.546) | -0.001 (0.678) | -0.008 (0.822) | 1.584 (1.055) | -0.915^*^ (0.489) | -0.786 (0.588) | 0.967 (0.624) | 2.179^***^ (0.800) |
| low increasing pattern | -3.142^****^ (0.898) | 1.329^*^ (0.716) | -4.194^***^ (1.353) | 1.161 (1.113) | -2.643^***^ (0.805) | 1.425^**^ (0.621) | -1.713^*^ (1.027) | -0.135 (0.845) |
| constant | 47.993^****^ (1.029) | 38.456^****^ (0.903) | 50.060^****^ (1.419) | 44.405^****^ (1.251) | 46.003^****^ (0.932) | 33.250^****^ (0.827) | 6.143^****^ (0.841) | 6.076^****^ (0.816) |
|  | | | | | | | | |
| Observations | 9,790 | 10,505 | 9,790 | 10,505 | 9,790 | 10,505 | 9,790 | 10,505 |
| R^2^ | 0.296 | 0.351 | 0.332 | 0.378 | 0.293 | 0.354 | 0.380 | 0.420 |
| Adjusted R^2^ | 0.296 | 0.350 | 0.332 | 0.378 | 0.293 | 0.354 | 0.380 | 0.419 |
| Residual Std. Error | 9.148 (df = 9784) | 9.254 (df = 10499) | 13.779 (df = 9784) | 14.391 (df = 10499) | 8.197 (df = 9784) | 8.024 (df = 10499) | 10.459 (df = 9784) | 10.917 (df = 10499) |
| F Statistic | 823.902^****^ (df = 5; 9784) | 1,133.655^****^ (df = 5; 10499) | 973.487^****^ (df = 5; 9784) | 1,278.581^****^ (df = 5; 10499) | 811.777^****^ (df = 5; 9784) | 1,150.498^****^ (df = 5; 10499) | 1,200.396^****^ (df = 5; 9784) | 1,518.531^****^ (df = 5; 10499) |
|  | | | | | | | | |
| *Note:* | .* p<0.05; ** p<0.01; *** p<0.001 | | | | | | | |

**Note: Abbreviations: SE, standard error; MAP, mean arterial pressure; SBP, systolic blood pressure; DBP, diastolic blood pressure,** Δ: changes**.** Hierarchical regression analysis results of the association between Mg intake classes (High decreasing vs normal stable and low increasing vs normal stable) and ΔBP (ΔMAP, ΔSBP, ΔDBP, ΔPP) at UK biobank study. Model 1 was **adjusted** for baseline BP and the main covariates including age, and education. Data represents unstandardized Beta correlation +/- SE (p). Beta is per one mg unit increment in Mg intake variables and expressed in SD units of the dependent variable**.**

**Table S10. Model 2 of the association between Mg intake trajectories over time and blood pressure changes at UK biobank study**

|  | **Δ MAP (mmHg)** | | **Δ SBP (mmHg)** | | **Δ DBP (mmHg)** | | **Δ PP (mmHg)** | |
| --- | --- | --- | --- | --- | --- | --- | --- | --- |
|  | **Men** | **Women** | **Men** | **Women** | **Men** | **Women** | **Men** | **Women** |
|  | **B (SE)** | **B (SE)** | **B (SE)** | **B (SE)** | **B (SE)** | **B (SE)** | **B (SE)** | **B (SE)** |
| High decreasing | -5.386^****^ (1.116) | -1.067 (1.743) | -4.903^***^ (1.682) | 1.905 (2.712) | -5.595^****^ (0.999) | -2.445 (1.512) | 0.779 (1.277) | 4.079^**^ (2.060) |
| low increasing | -3.778^***^ (1.154) | 1.381 (0.881) | -5.268^***^ (1.740) | 1.345 (1.371) | -3.113^***^ (1.033) | 1.424^*^ (0.764) | -2.324^*^ (1.322) | 0.080 (1.041) |
| Baseline Mg | 0.001 (0.001) | -0.001 (0.001) | 0.002 (0.001) | -0.003^*^ (0.001) | 0.001 (0.001) | -0.001 (0.001) | 0.001 (0.001) | -0.001 (0.001) |
| Baseline Mg x High decreasing | 0.011^****^ (0.003) | 0.004 (0.004) | 0.011^***^ (0.004) | 0.002 (0.007) | 0.012^****^ (0.002) | 0.005 (0.004) | -0.001 (0.003) | -0.003 (0.005) |
| Baseline Mg x low increasing | 0.004 (0.005) | 0.001 (0.005) | 0.006 (0.007) | 0.001 (0.008) | 0.003 (0.004) | 0.001 (0.004) | 0.003 (0.006) | -0.0004 (0.006) |
| Constant | 48.567^****^ (1.046) | 39.303^****^ (0.921) | 50.376^****^ (1.440) | 45.236^****^ (1.272) | 46.616^****^ (0.946) | 33.912^****^ (0.844) | 6.065^****^ (0.847) | 6.096^****^ (0.824) |
|  | | | | | | | | |
| Observations | 9,790 | 10,505 | 9,790 | 10,505 | 9,790 | 10,505 | 9,790 | 10,505 |
| R^2^ | 0.299 | 0.352 | 0.333 | 0.380 | 0.296 | 0.355 | 0.380 | 0.420 |
| Adjusted R^2^ | 0.298 | 0.352 | 0.333 | 0.379 | 0.295 | 0.355 | 0.380 | 0.419 |
| Residual Std. Error | 9.135 (df = 9780) | 9.243 (df = 10495) | 13.771 (df = 9780) | 14.379 (df = 10495) | 8.182 (df = 9780) | 8.017 (df = 10495) | 10.459 (df = 9780) | 10.917 (df = 10495) |
| F Statistic | 462.783^****^ (df = 9; 9780) | 634.606^****^ (df = 9; 10495) | 543.171^****^ (df = 9; 9780) | 714.138^****^ (df = 9; 10495) | 457.154^****^ (df = 9; 9780) | 642.583^****^ (df = 9; 10495) | 667.144^****^ (df = 9; 9780) | 844.074^****^ (df = 9; 10495) |
|  | | | | | | | | |
| *Note:* | .* p<0.05; ** p<0.01; *** p<0.001 | | | | | | | |

**Note: Abbreviations: SE, standard error; MAP, mean arterial pressure; SBP, systolic blood pressure; DBP, diastolic blood pressure,** Δ: changes**.** Hierarchical regression analysis results of the association between Mg intake classes (High decreasing vs normal stable and low increasing vs normal stable) and ΔBP (ΔMAP, ΔSBP, ΔDBP, ΔPP) at UK biobank study. Model 1 was **adjusted** for baseline BP and the main covariates including age, and education. Model 2 was additionally tested the two-way interactions between baseline Mg x Mg classes while controlling for **baseline** Mg **and** antihypertensive medication. Data represents unstandardized Beta correlation +/- SE (p). Beta is per one mg unit increment in Mg intake variables and expressed in SD units of the dependent variable**.**

**Table S11. Model 3 of the association between Mg intake trajectories over time and blood pressure changes at UK biobank study**

|  | | | | | | | | |
| --- | --- | --- | --- | --- | --- | --- | --- | --- |
|  | **Δ MAP (mmHg)** | | **Δ SBP (mmHg)** | | **Δ DBP (mmHg)** | | **Δ PP (mmHg)** | |
|  | **Men** | **Women** | **Men** | **Women** | **Men** | **Women** | **Men** | **Women** |
|  | **B (SE)** | **B (SE)** | **B (SE)** | **B (SE)** | **B (SE)** | **B (SE)** | **B (SE)** | **B (SE)** |
| High decreasing | -5.386^****^ (1.116) | -1.067 (1.743) | -4.903^***^ (1.682) | 1.905 (2.712) | -5.595^****^ (0.999) | -2.445 (1.512) | 0.779 (1.277) | 4.079^**^ (2.060) |
| low increasing | -3.778^***^ (1.154) | 1.381 (0.881) | -5.268^***^ (1.740) | 1.345 (1.371) | -3.113^***^ (1.033) | 1.424^*^ (0.764) | -2.324^*^ (1.322) | 0.080 (1.041) |
| Baseline Mg | 0.001 (0.001) | -0.001 (0.001) | 0.002 (0.001) | -0.003^*^ (0.001) | 0.001 (0.001) | -0.001 (0.001) | 0.001 (0.001) | -0.001 (0.001) |
| Baseline Mg x High decreasing | 0.011^****^ (0.003) | 0.004 (0.004) | 0.011^***^ (0.004) | 0.002 (0.007) | 0.012^****^ (0.002) | 0.005 (0.004) | -0.001 (0.003) | -0.003 (0.005) |
| Baseline Mg x low increasing | 0.004 (0.005) | 0.001 (0.005) | 0.006 (0.007) | 0.001 (0.008) | 0.003 (0.004) | 0.001 (0.004) | 0.003 (0.006) | -0.0004 (0.006) |
| Constant | 48.567^****^ (1.046) | 39.303^****^ (0.921) | 50.376^****^ (1.440) | 45.236^****^ (1.272) | 46.616^****^ (0.946) | 33.912^****^ (0.844) | 6.065^****^ (0.847) | 6.096^****^ (0.824) |
| Observations | 9,790 | 10,505 | 9,790 | 10,505 | 9,790 | 10,505 | 9,790 | 10,505 |
| R^2^ | 0.299 | 0.352 | 0.333 | 0.380 | 0.296 | 0.355 | 0.380 | 0.420 |
| Adjusted R^2^ | 0.298 | 0.352 | 0.333 | 0.379 | 0.295 | 0.355 | 0.380 | 0.419 |
| Residual Std. Error | 9.135 (df = 9780) | 9.243 (df = 10495) | 13.771 (df = 9780) | 14.379 (df = 10495) | 8.182 (df = 9780) | 8.017 (df = 10495) | 10.459 (df = 9780) | 10.917 (df = 10495) |
| F Statistic | 462.783^****^ (df = 9; 9780) | 634.606^****^ (df = 9; 10495) | 543.171^****^ (df = 9; 9780) | 714.138^****^ (df = 9; 10495) | 457.154^****^ (df = 9; 9780) | 642.583^****^ (df = 9; 10495) | 667.144^****^ (df = 9; 9780) | 844.074^****^ (df = 9; 10495) |
| *Note:* | .* p<0.05; ** p<0.01; *** p<0.001 | | | | | | | |

**Note: Abbreviations: SE, standard error; MAP, mean arterial pressure; SBP, systolic blood pressure; DBP, diastolic blood pressure,** Δ: changes**.** Hierarchical regression analysis results of the association between Mg intake trajectories (“High decreasing” vs “normal stable” and “low increasing” vs “normal stable”) and ΔBP (ΔMAP, ΔSBP, ΔDBP, ΔPP) at UK biobank study. Model 1 was **adjusted** for baseline BP and the main covariates including age, and education. Model 2 was additionally tested the two-way interactions between baseline Mg x Mg trajectories while controlling for **baseline** Mg **and** antihypertensive medication. **Model 3 was** additionally **adjusted for main covariates: HDL, cholesterol, diabetes mellitus, smoking status, higher education, physical activity, and alcohol intake.** Data represents unstandardized Beta correlation +/- SE (p). Beta is per one mg unit increment in Mg intake variables and expressed in SD units of the dependent varia


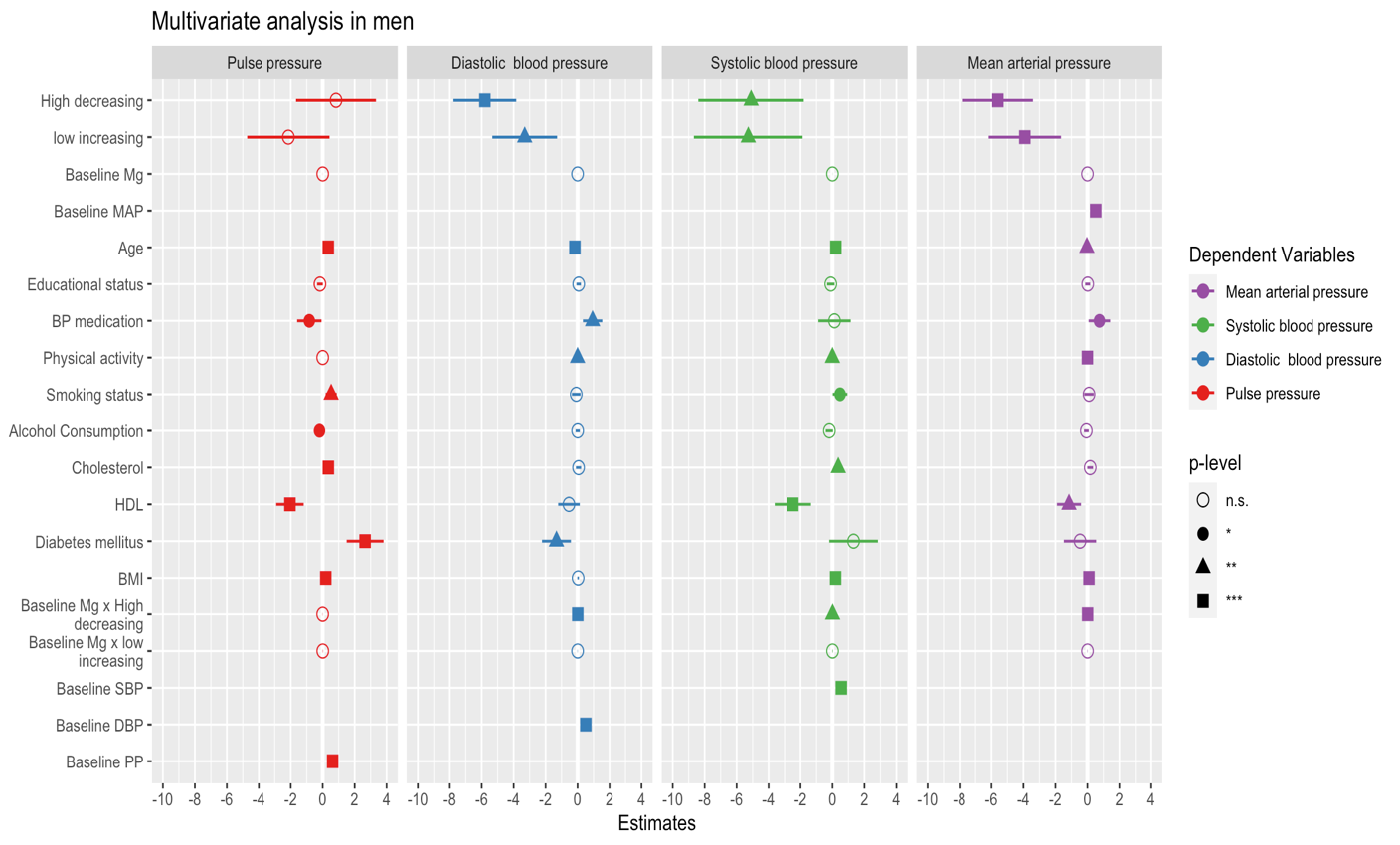

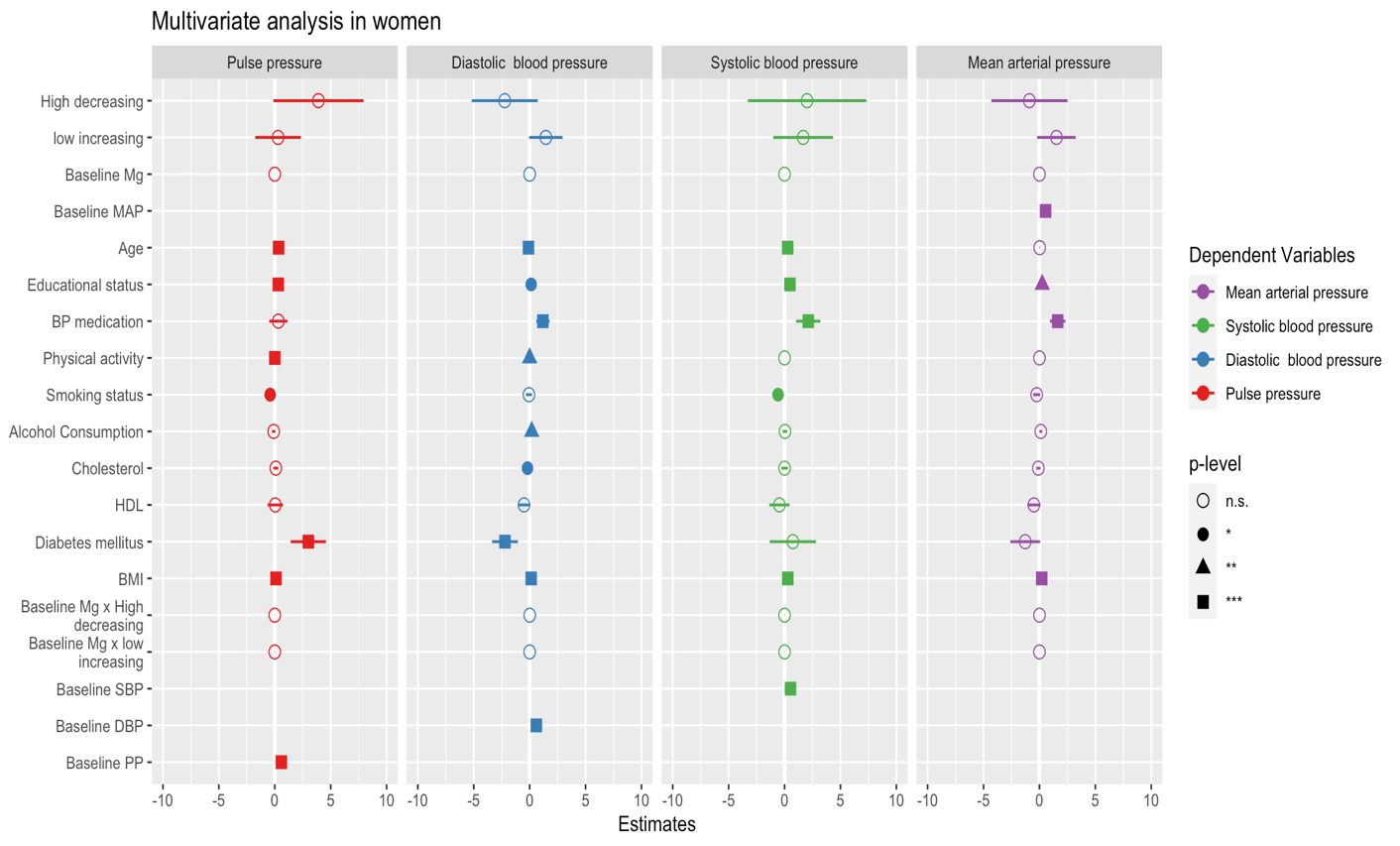


**Figure S9 Hierarchical regression analysis results of the association between Magnesium intake classes and changes in blood pressure measures including mean arterial pressure, systolic blood pressure, diastolic blood pressure and pulse pressure, at UK biobank study.** **Model 3 was adjusted for Baseline, Mg x classes, both baseline BP and Mg and main covariates: HDL, cholesterol, diabetes, smoking status, higher education, physical activity, alcohol intake, and antihypertensive medication. Note. Error bars represent standard error from the same model. Significance is at a < 0.05.**

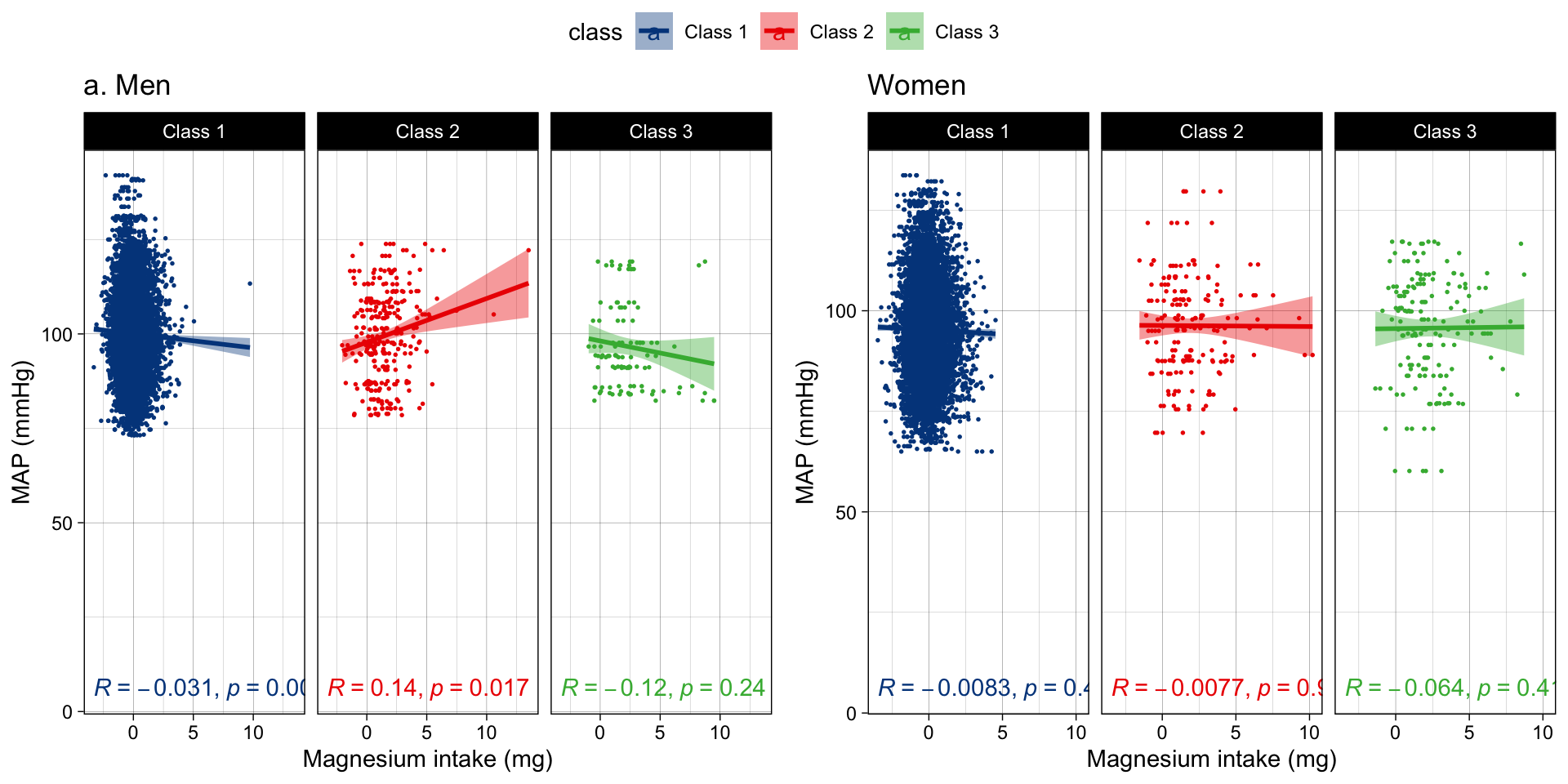


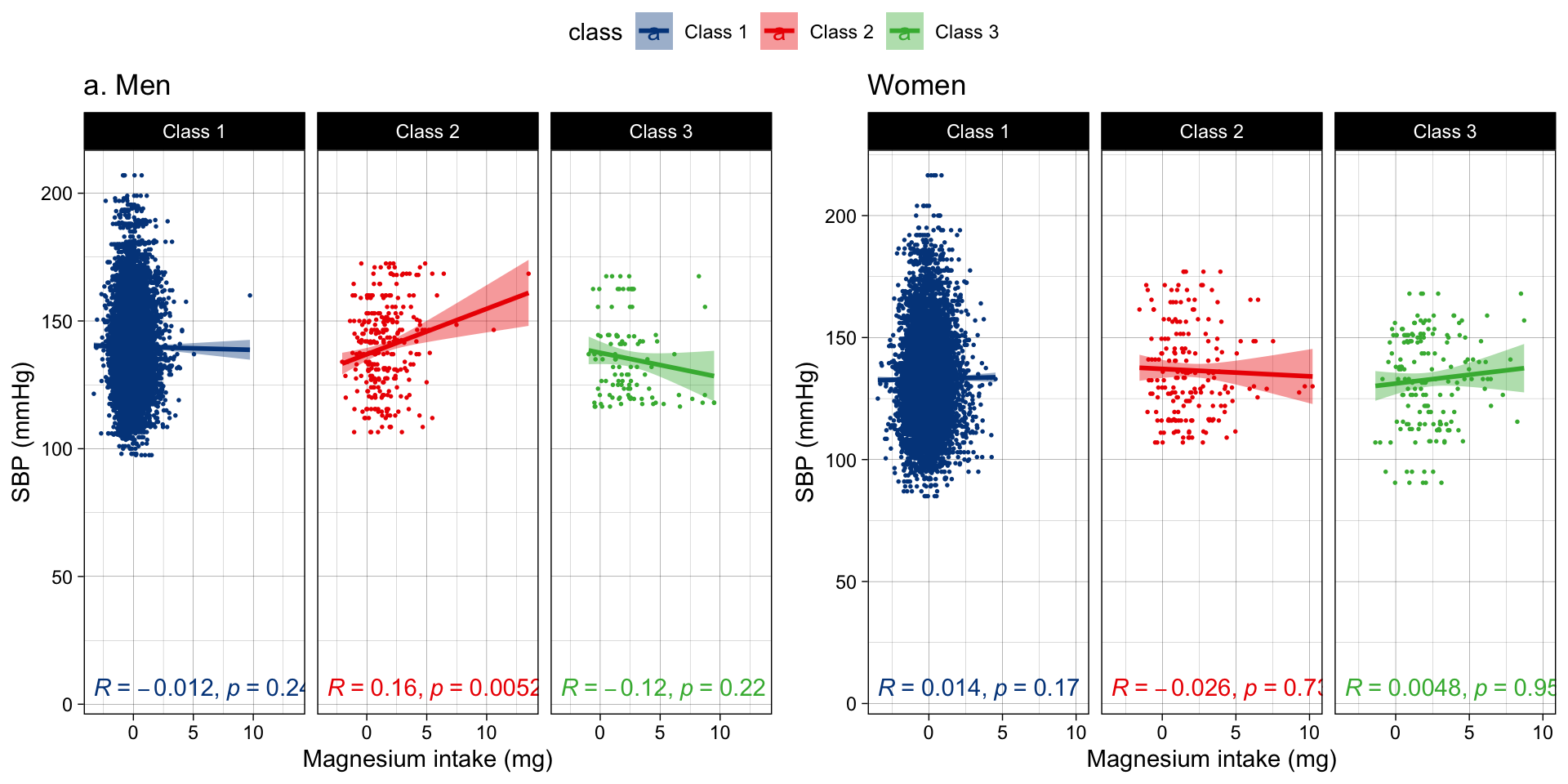


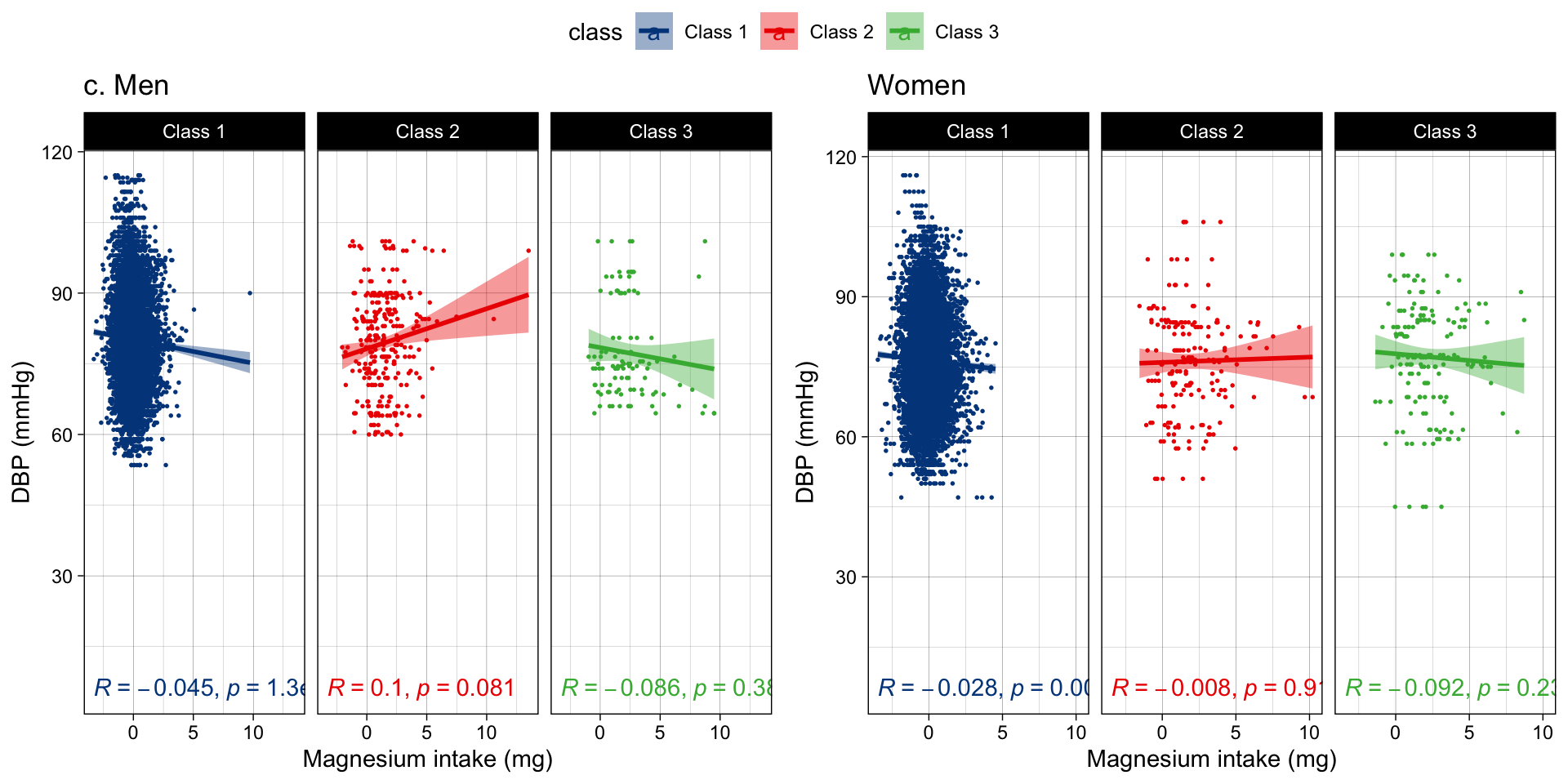


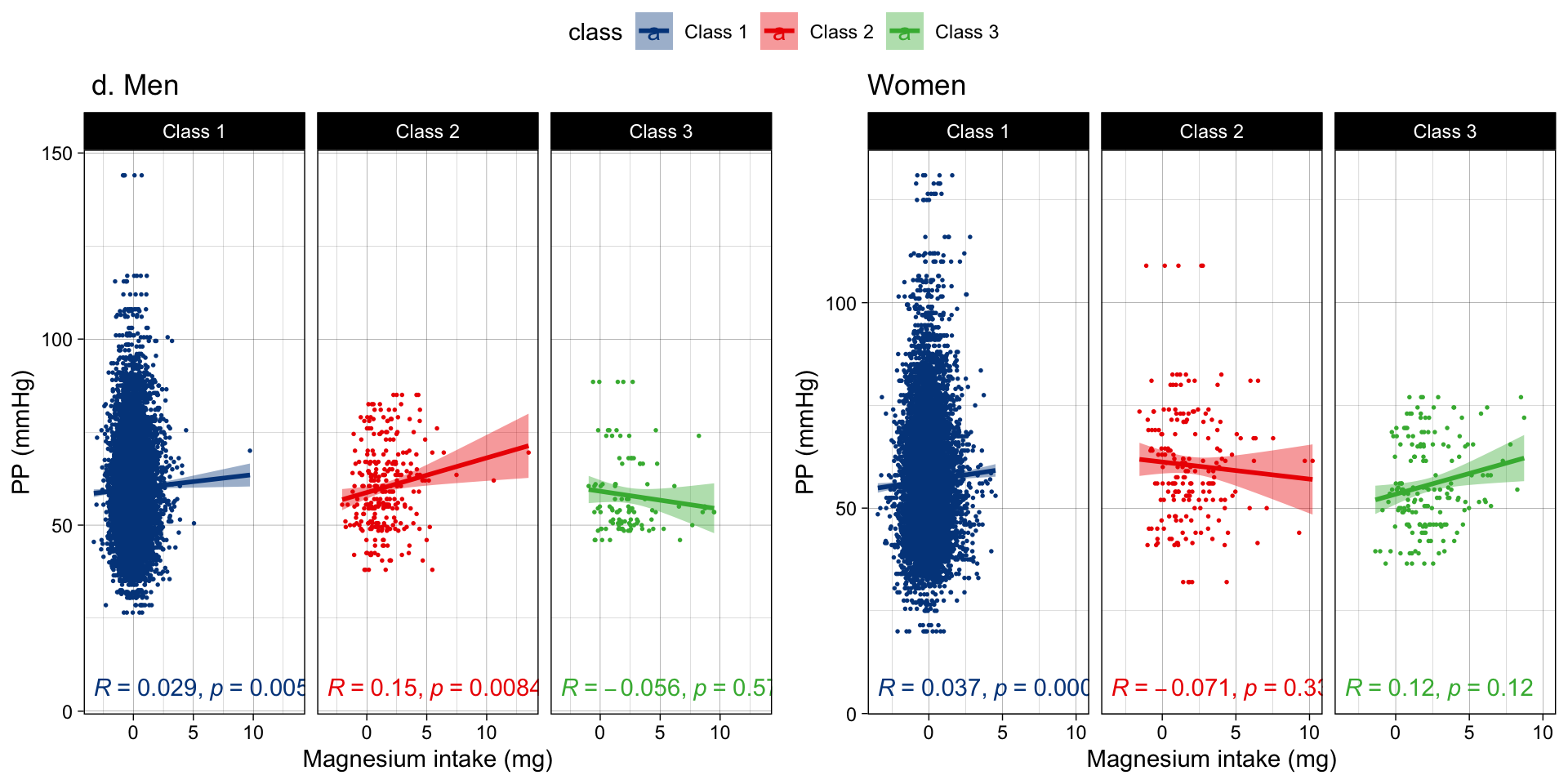


**Figure S10 the scatter plots show the association direction between Magnesium intake classes (class1 [normal stable], class 2 [high decreasing], class 3[low increasing]) and blood pressure changes including** A) mean arterial pressure (MAP); B) systolic blood pressure (SBP), C) diastolic blood pressure (DBP); D) pulse pressure (PP) in men and women **at UK biobank study.** **Model 3 was assessed the two-way interaction between baseline Mg and Mg trajectories and adjusted for baseline BP and main covariates: age, HDL, cholesterol, diabetes, smoking status, higher education, physical activity, alcohol intake, and antihypertensive medication.** **Significance is at a < 0.05.**

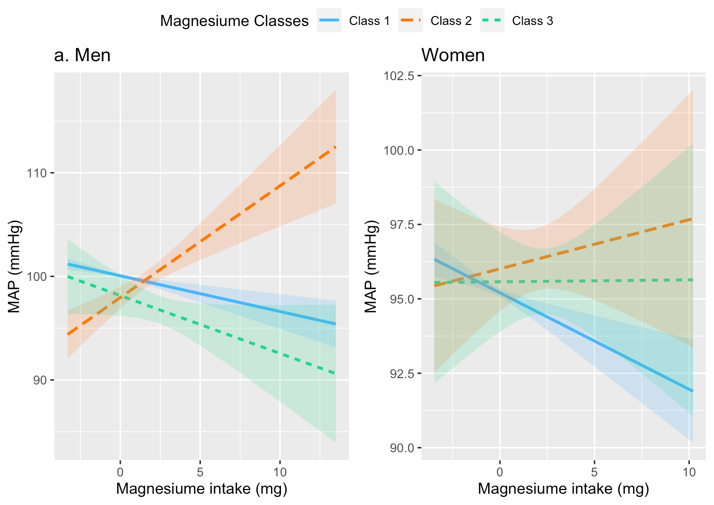

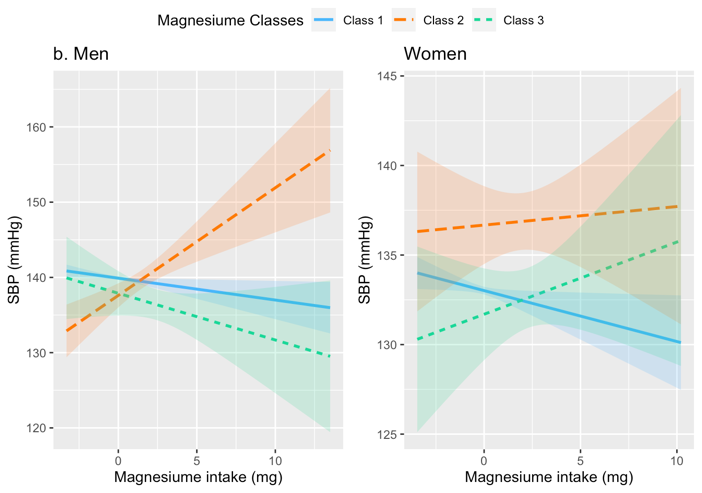


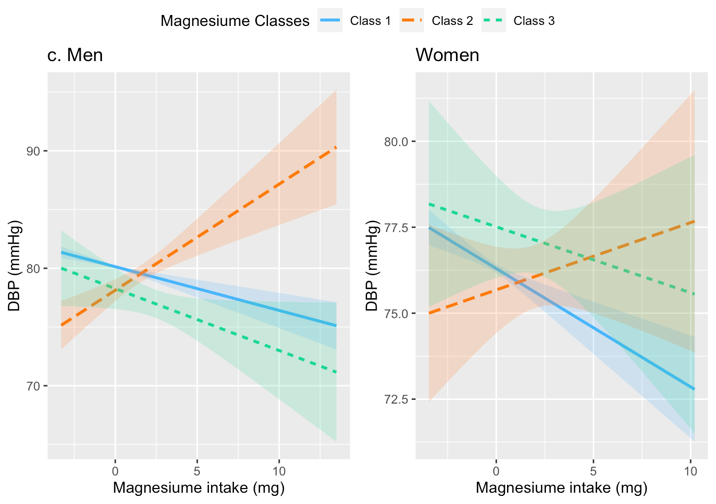

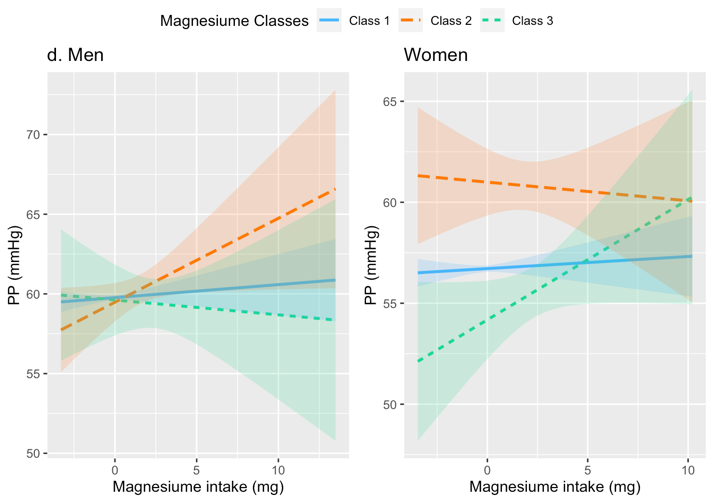


**Figure S11**. Two-way interaction between baseline Mg and Mg classes and BP changes including a) mean arterial pressure (MAP); b) systolic blood pressure (SBP), c) diastolic blood pressure (DBP); d) pulse pressure (PP) in men and women.

**Table S12.** Women’ demographic characteristics.

| **Measures** | **Whole Sample** | **Pre menopause** | **Post menopause** | **T/chi-sq Test (P value)** |
| --- | --- | --- | --- | --- |
| Age, year (SD) | 54.70 (7.34) | 47.12 (4.24) | 58.50 (5.34) | -145.50 (0.000) |
| SBP (wave 1), mmHg (SD) | 131.58 (18.37) | 125.63 (16.42) | 134.56 (18.57) | -30.78 (0.000) |
| DBP (wave 1), mmHg (SD) | 79.30 (9.68) | 78.30 (9.80) | 79.80 (9.57) | -9.15 (0.000) |
| BMI, kg/m2 (SD) | 25.99 (4.40) | 25.68 (4.49) | 26.14 (4.34) | -6.05 (0.000) |
| Cholesterol, mmol/L, (SD) | 5.86 (1.06) | 5.44 (0.94) | 6.07 (1.05) | -37.86 (0.000) |
| HDL mmol/L, (SD) | 1.64 (0.36) | 1.59 (0.33) | 1.67 (0.37) | -13.52 (0.000) |
| GM, mm3 (SD) | 637901.28 (46558.86) | 651873.02 (44857.73) | 630908.76 (45816.47) | 27.47 (0.000) |
| WM volume, mm3 (SD) | 452577.18 (45441.18) | 461276.20 (44587.36) | 448223.52 (45240.82) | 17.25 (0.000) |
| Left HC volume, mm3 (SD) | 3574.77 (350.11) | 3689.95 (319.06) | 3517.12 (350.75) | 31.01 (0.000) |
| Right HC volume, mm3 (SD) | 3684.01 (352.18) | 3790.96 (324.28) | 3630.48 (353.41) | 28.42 (0.000) |
| WMLs volume, mm3 (SD) | 7.26 (0.64) | 6.98 (0.46) | 7.39 (0.67) | -45.47 (0.000) |
| ICV volume, mm3 (SD) | 1472982.76 (116580.53) | 1492135.51 (114726.53) | 1463397.27 (116324.65) | 14.76 (0.000) |
| Magnesium (wave 1), mg (SD) | 342.34 (115.83) | 340.67 (116.52) | 343.18 (115.48) | -1.28 (0.200) |
| Magnesium (wave 2), mg (SD) | 338.08 (89.88) | 331.77 (88.16) | 341.23 (90.57) | -6.30 (0.000) |
| Magnesium (wave 3), mg (SD) | 336.23 (88.55) | 333.43 (91.88) | 337.63 (86.81) | -2.76 (0.006) |
| Magnesium (wave 4), mg (SD) | 334.85 (94.77) | 329.28 (89.67) | 337.63 (97.11) | -5.36 (0.000) |
| Magnesium (wave 5), mg (SD) | 338.38 (90.49) | 337.13 (93.09) | 339.00 (89.16) | -1.21 (0.227) |
| Hypertension, n (%) | 5345 (33.83%) | 1135 (21.66%) | 4180 (39.81%) | 514.02 (0.000) |
| BP medication, n (%) | 1035 (6.56%) | 210 (3.99%) | 825 (7.85%) | 84.43 (0.000) |
| Diabetes, n (%) | 340 (2.16%) | 70 (1.33%) | 270 (2.57%) | 24.89 (0.000) |
| Ever smoked, n (%) | 10350 (65.63%) | 3755 (71.39%) | 6595 (62.75%) | 115.58 (0.000) |
| Higher Education, n (%) | 7400 (46.92%) | 2715 (51.62%) | 4685 (44.58%) | 69.47 (0.000) |
| Significance: p<0.05 | | | | |

**Abbreviations: SBP; systolic blood pressure, DBP; diastolic blood pressure, MAP; mean arterial pressure, PP; pulse pressure, GM; gray matter, HC; hippocampus, WMLs, white matter lesions, ICV; intracranial volume, BMI; body mass index. Significance: p<0.05**


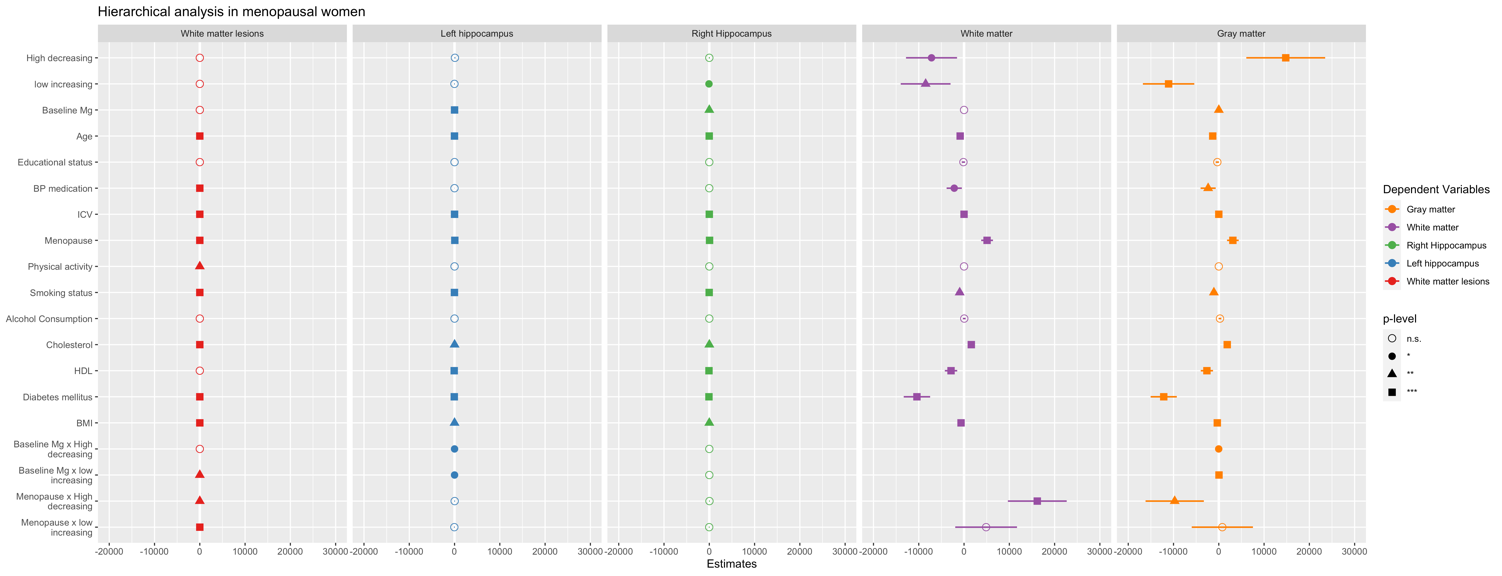


**Figure S12. Hierarchical regression analysis results of the association between Magnesium intake classes and brain volumes including gray matter, left hippocampal, right hippocampal, white matter lesions at UK biobank study.** Model 1 was **adjusted** for the main covariates including age, education and **ICV.** Model 2 was additionally tested the two-way interactions between baseline Mg x Mg trajectories while controlling for **baseline** Mg **and** antihypertensive medication. **Model 3 was** additionally tested the two-way interactions between menopausal status x Mg trajectories while controlling **for the other covariates: age, education and antihypertensive medication HDL, cholesterol, diabetes mellitus, smoking status, higher education, physical activity, and alcohol intake. Note. Error bars represent standard error from the same model.**

**
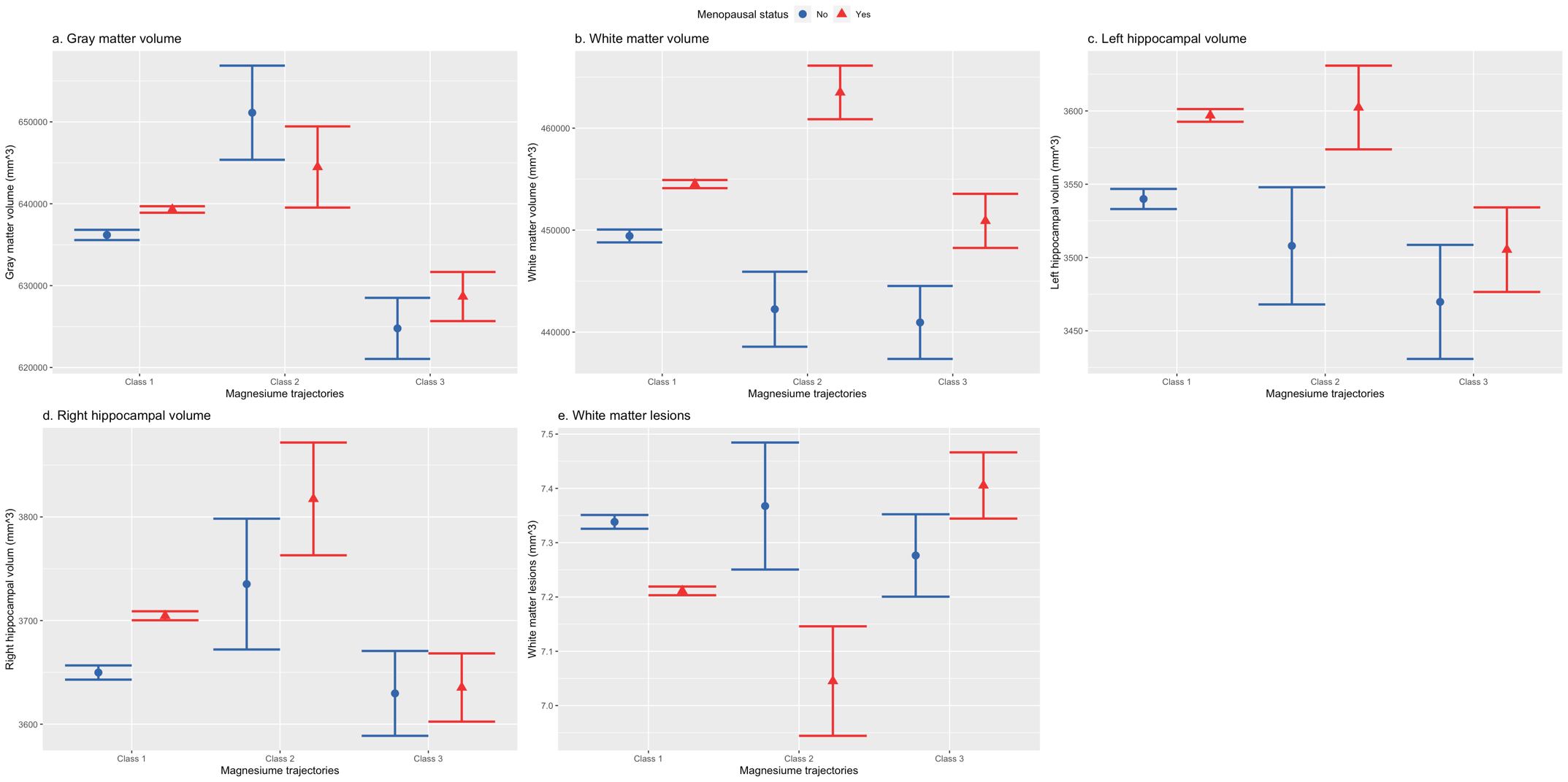
**

**Figure S13.** The interaction effects between Mg intake trajectories (“High decreasing” vs “normal stable” and “low increasing” vs” normal stable”) and menopausal status (pre menopause [blue colour] vs post menopause [red colour]) in predicating brain volumes including a) gray matter; b) white matter; c) left hippocampal volume; d) right hippocampal volume; e) white matter lesions, in women. The bars indicated the 95% confidence intervals (CI).
